# Chronic expression of p16^INK4a^ in the epidermis induces Wnt-mediated hyperplasia and promotes tumor initiation

**DOI:** 10.1038/s41467-020-16475-3

**Published:** 2020-06-01

**Authors:** Narmen Azazmeh, Benjamin Assouline, Eitan Winter, Shmuel Ruppo, Yuval Nevo, Alexander Maly, Karen Meir, Agnieszka K. Witkiewicz, Jonathan Cohen, Sophia V. Rizou, Eli Pikarsky, Chen Luxenburg, Vassilis G. Gorgoulis, Ittai Ben-Porath

**Affiliations:** 1 Department of Developmental Biology and Cancer Research, Institute for Medical Research – Israel-Canada, The Hebrew University–Hadassah Medical School, Jerusalem, Israel; 2 Info-CORE, Bioinformatics Unit of the I-CORE Computation Center at the Hebrew University and Hadassah, Jerusalem, Israel; 3 Department of Pathology, Hadassah-Hebrew University Medical Center, Jerusalem, Israel; 4 Roswell Park Cancer Institute, Elm and Carlton Streets, Buffalo, NY USA; 5 Department of Cell and Developmental Biology, Sackler Faculty of Medicine Tel Aviv University, Tel Aviv, Israel; 6 Department of Histology and Embryology, Medical School, National and Kapodistrian University of Athens, Athens, Greece; 7 The Lautenberg Center for Immunology and Cancer Research, Hebrew University of Jerusalem Faculty of Medicine, Jerusalem, Israel; 8 Faculty Institute for Cancer Sciences, Manchester Academic Health Sciences Centre, University of Manchester, Manchester, UK; 9 Biomedical Research Foundation, Academy of Athens, Athens, Greece; 10 Center for New Biotechnologies and Precision Medicine, Medical School, National and Kapodistrian University of Athens, Athens, Greece

**Keywords:** Cancer models, Skin cancer, Tumour-suppressor proteins

## Abstract

p16^INK4a^ (CDKN2A) is a central tumor suppressor, which induces cell-cycle arrest and senescence. Cells expressing p16^INK4a^ accumulate in aging tissues and appear in premalignant lesions, yet their physiologic effects are poorly understood. We found that prolonged expression of transgenic p16^INK4a^ in the mouse epidermis induces hyperplasia and dysplasia, involving high proliferation rates of keratinocytes not expressing the transgene. Continuous p16^INK4a^ expression increases the number of epidermal papillomas formed after carcinogen treatment. Wnt-pathway ligands and targets are activated upon prolonged p16^INK4a^ expression, and Wnt inhibition suppresses p16^INK4a^-induced hyperplasia. Senolytic treatment reduces p16^INK4a^-expressing cell numbers, and inhibits Wnt activation and hyperplasia. In human actinic keratosis, a precursor of squamous cell carcinoma, p16^INK4a^-expressing cells are found adjacent to dividing cells, consistent with paracrine interaction. These findings reveal that chronic p16^INK4a^ expression is sufficient to induce hyperplasia through Wnt-mediated paracrine stimulation, and suggest that this tumor suppressor can promote early premalignant epidermal lesion formation.

## Abstract (web summary)

It is unclear how resident p16-expressing senescent cells affect the propensity of tissues to develop cancer. Here, the authors show that chronic p16 expression in the mouse epidermis causes hyperplasia and dysplasia through Wnt-mediated paracrine stimulation of proliferating keratinocytes, and can contribute to tumour formation.

## Introduction

Activity of tumor-suppressive cellular mechanisms is often associated with cell and organismal aging, yet cancer propensity substantially increases with age^1–3^. p16^INK4a^, encoded by the *CDKN2A* gene (p16 hereafter), represents an important link between cancer, cellular responses to stress, and aging. p16 is a central tumor suppressor, which is among the most commonly mutated genes in diverse human malignancies^4,5^. When activated, p16 binds and inhibits CDK4/6-Cyclin D complexes, leading to Rb activation, and thereby induces cell-cycle arrest and senescence^4,6^. This pathway represents one of the central mechanisms blocking the proliferation of damaged or oncogene-expressing cells.

Whereas p16 is not expressed in most embryonic and adult cells^7^, its levels increase in multiple tissues with age^8–11^. The specific stimuli underlying age-associated p16 activation have not been directly established. However, a variety of stresses, including radiation, DNA damaging agents, cigarette smoke, and oncogene activity, were shown to induce p16^12–15^. Aged animals lacking p16 show increased replicative and regenerative capacity in several tissues, indicating that it contributes to the aging-associated decline in these processes^1^. It was more recently shown that directed genetic elimination of p16-expressing senescent cells during mouse aging delays the functional deterioration of multiple organs and increases lifespan^11^. This finding and subsequent studies have highlighted the negative contribution of senescent cells to age-associated pathologies, and the therapeutic potential for their pharmacologic removal through senolytic drug treatment^16,17^. Whether senolytic treatments have potential benefit in cancer therapy is currently largely unknown.

The expression of p16 in aging tissues raises the question of whether its activity influences cancer development. Mice carrying an extra copy of *Cdkn2a* show increased resistance to cancer, consistent with the known tumor-suppressive role of p16^18^. In contrast, elimination of p16-expressing senescent cells reduces cancer mortality rates in mice, suggesting that such cells could contribute to tumor development^11^. The mechanisms underlying this are not fully known. It has been suggested that resident senescent cells can promote tumorigenesis during aging by generating inflammation mediated by cytokine secretion, a feature of senescence known as the senescence-associated secretory phenotype (SASP)^3,19^. It is, however, unclear whether all cells expressing p16 in vivo achieve a full senescence phenotype, and p16 activity itself appears to be insufficient to induce the SASP^20,21^. The functional contributions of p16 to age-associated changes in cancer propensity, therefore, remain poorly characterized.

Here we study the effects of prolonged p16 expression in the epidermis, in order to uncover its effects on tissue structure and cancer development. p16 levels and senescence were reported to increase with age in the skin dermis and epidermis^22–24^. UV radiation (UVR), the major cause of skin malignancies, activates p16^13,25^, and p16-expressing cells are detected in premalignant epidermal lesions such as actinic keratosis^26–28^. The high mutation rates of p16 in cutaneous squamous cell carcinoma and other skin malignancies^5,29,30^ indicate that it suppresses malignant progression. However, it is unknown whether the activity of p16 in the normal epidermis and in premalignant lesions influences the development of disease. Furthermore, whether p16-expressing cells in such early lesions can be targeted by senolytic therapy, and whether this may have therapeutic benefit, has not been tested.

Using transgenic mice allowing tissue-specific p16 activation, we demonstrate that the persistent expression of p16 in a subset of cells within the epidermis induces hyperplasia and dysplasia, and promotes tumor formation following mutagenesis. We show that p16 expression in mice and in cultured keratinocytes leads to Wnt-pathway activation, which contributes to epidermal hyperproliferation, and that senolytic elimination of p16-expressing cells inhibits hyperplasia. These findings reveal that chronic p16 activity is sufficient to induce premalignant tissue changes through a non-cell-autonomous mechanism, and uncover a potential tumor-promoting function of this gene during early tumorigenesis.

## Results

### Epidermal p16 induction causes partial senescence features

**Fig. 1:**
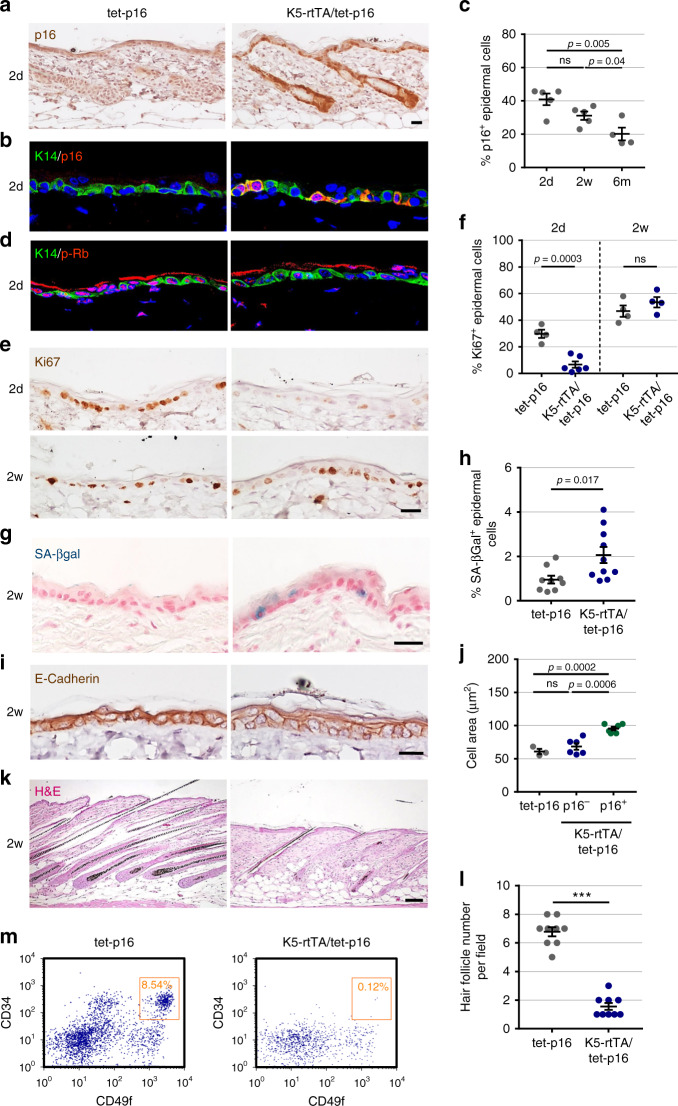
p16 induces cell-cycle arrest and cell hypertrophy, and disrupts hair-follicle growth. **a** Immunostaining of human p16 (brown) in back skin sections of K5-rtTA/tet-p16 and control tet-p16 mice treated with doxycycline (dox) for 2 days (2d) starting at 3 weeks of age. **b** Skin sections from the same mice stained for p16 (red) and K14 (green), which labels the basal epidermis. **c** Percentage of p16^+^ keratinocytes in the interfollicular epidermis (IFE) in mice treated with dox for 2 days (2d), 2 weeks (2w), or 6 months (6 m). Dots indicate individual mice, *n* = 5, 5, 4 in respective groups. **d** Skin sections from mice treated with dox for 2 days, stained for Rb phosphorylated on Thr-821/826 (p-Rb) (red) and K14 (green). **e** Immunostaining of the proliferation marker Ki67 (brown) in sections from mice treated with dox for 2 days (2d) or 2 weeks (2w). **f** Percentage of IFE keratinocytes expressing Ki67 in the same mice (dots). *n* = 4, 6, 4, 4. **g** SA-βGal staining (blue) of skin sections from indicated mice, treated with dox for 2 weeks. **h** Percentage of SA-βGal positive IFE cells from indicated mice (dots) treated with dox for 2 weeks. *n* = 9, 10. **i** Skin sections from indicated mice stained for E-Cadherin (brown) to indicate cell circumference. **j** Area of epidermal keratinocytes from indicated mice (dots). Shown are values for control mice (tet-p16), and for p16^−^ and p16^+^ keratinocytes in K5-rtTA/tet-p16 mice. *n* = 3, 6, 6 mice, >60 cells were scored per mouse. **k** H&E-stained skin sections from the indicated mice treated with dox for 2 weeks, showing hair-follicle morphology. **l** Number of hair follicles per field in indicated mice (dots), following dox treatment for 6 months. *n* = 9 per group. **m** FACS analysis of epidermal cells from indicated mice treated with dox for 6 months, stained for CD34, CD49f, and Sca1. Charts show Sca1^−^ (follicular) cells only. Gate indicates percentage of CD34^+^/CD49f^high^ hair-follicle stem cells. Cells were pooled from three mice per group. All graphs indicate mean across mice ± S.E.M, all scores were conducted visually from images. Blue labels DNA in all fluorescence images. ***P < 0.0001, *t* test. ns non-significant. Scale bars—20 μm, except **k**—100 μm.

To study the effects of p16-expressing cells on the adult skin we crossed mice carrying a doxycycline-activated human p16 gene (tet-p16)^21^ with K5-rtTA mice^31^, allowing its inducible activation in the basal epidermis. Transgenic p16 protein was detected in ~40% of basal keratinocytes in the interfollicular epidermis (IFE) after 2 days of doxycycline (dox) treatment at 3 weeks of age (Fig. 1a–c). Tissues showed reduced phosphorylated Rb levels, consistent with expected Rb activation (Fig. 1d). Staining for Ki67 revealed a dramatic reduction in the number of proliferating IFE basal cells after 2 days of p16 activation (Fig. 1e, f). However, after 2 weeks of p16 activation, overall proliferation rates returned to those of control animals.

Increased numbers of cells expressing the senescence markers SA-βGal and SenTraGor^32^ were detected in mice that have undergone 2 weeks of p16 activation (Fig. 1g, h and Supplementary Fig. 1a, b), yet those were lower than the numbers of p16-expressing cells, suggesting that not all transgene-expressing cells enter a fully senescent state. The average size of keratinocytes was increased in p16-expressing mice, specifically cells expressing the transgene, resulting in a thickening of the epidermis, with no increase in cell numbers (Fig. 1i, j and Supplementary Fig. 1c–e). Changes in other known markers of senescence such as p21, Lamin B1, DNA damage, or secreted factors were not detected. Together these findings indicate that p16 expression in epidermal keratinocytes induces cell-cycle arrest, cell hypertrophy, and some features of senescence.

p16 induction led to delayed growth of hair follicles (HF), reduced hair-follicle stem cell (HFSC) proliferation, and the formation of deformed HFs during the anagen stage initiated at 3 weeks of age (Fig. 1k and Supplementary Fig. 1f, g), reminiscent of the effect of p14^ARF^ activation^33^. Upon prolonged p16 expression, mice displayed a decrease in the number of HFs and hair loss (Fig. 1l and Supplementary Fig. 1f, h), and the numbers of CD34^+^/CD49f^high^ HFSCs were dramatically reduced (Fig. 1m). p16 expression thus blocks the proliferation of HFSCs and leads to their gradual depletion.

### Prolonged p16 expression induces hyperplasia and dysplasia

**Fig. 2:**
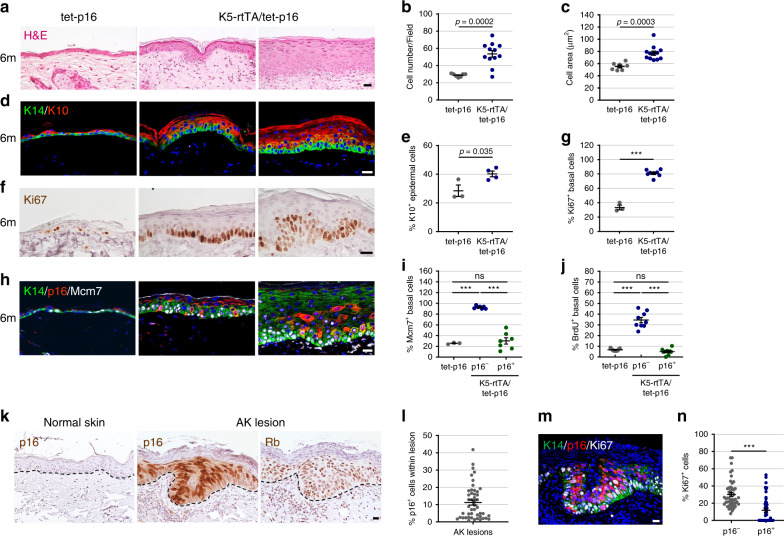
Chronic p16 expression causes epidermal hyperplasia and dysplasia. **a** H&E-stained skin sections of K5-rtTA/tet-p16 and control tet-p16 mice treated with dox for 6 months (6m). Typical (middle) and pronounced (right) phenotype regions are shown for p16-induced mice. **b** IFE keratinocyte cell number per field (40×) in indicated mice (dots). *n* = 7, 12 in respective groups. **c** Average area of IFE cells from the same mice, visually measured from E-cadherin-stained sections. *n* = 7, 12. **d** Sections from the same mice stained for K10 (red) labeling suprabasal cells and K14 (green) labeling basal cells. **e** Percentage of K10^+^ epidermal cells isolated from dissociated mouse ear epidermis of indicated mice (dots) after 6 months of dox treatment, scored by FACS. *n* = 3, 4. **f** Skin sections from the indicated mice stained for Ki67 (brown). Right image shows a region with dysplasia. **g** Percentage of Ki67^+^ basal IFE cells in indicated mice (dots) after 6 months of dox treatment. *n* = 3, 8. **h** Skin sections from same mice co-stained for human p16 (red), the proliferation marker Mcm7 (white) and K14 (green). **i** Percentage of Mcm7^+^ basal IFE cells in control mice (tet-p16), and among p16^−^ and p16^+^ cells in the epidermis of K5-rtTA/tet-p16 mice treated with dox for 6 months. *n* = 3, 7, 7. **j** Percentage of BrdU^+^ basal epidermal cells in same mice, scored from skin sections co-stained for BrdU and p16. *n* = 9 per group. **k** Serial sections of a human actinic keratosis lesion stained for p16 (middle) and Rb (right) (both brown). Staining for p16 in normal human skin is shown in left panel. Dotted lines label the basement membrane. **l** Percentage of p16^+^ cells within individual human actinic keratosis (AK) lesions (dots), among lesions showing p16 expression. *n* = 47. **m** AK lesion co-stained for p16 (red), Ki67 (white) and K14 (green). **n** Percentage of Ki67^+^ cells among p16^−^ and p16^+^ cells in human AK lesions (dots). *n* = 47. All graphs indicate mean across mice ± S.E.M, except panels **l**, **n** which indicate mean across lesions. All scores were conducted visually on images, except **e**. Blue labels DNA in all images. ***P < 0.0001, *t* test. ns non-significant. Scale bars—20 μm.

To examine the effects of prolonged p16 expression on the epidermis, we activated the transgene for 6 months. At this timepoint we detected p16 protein in ~20% of IFE cells (Fig. 1c). Strikingly, p16-expressing mice exhibited a thickened epidermis, in which both cell number and average cell size were increased (Fig. 2a–c). We observed an increased proportion of suprabasal differentiated cells in p16-expressing skins, while basal layer marker expression (K14, K5) was maintained and expanded in some regions (Fig. 2a, d, e and Supplementary Fig. 2a, b). These changes contrast with the normal young and aged mouse epidermis, which contains only a thin suprabasal layer (Supplementary Fig. 2c). p16-induced mice also often showed an expansion of the dermis with dense collagen deposition regions, and a reduction of fat layer width (Supplementary Fig. 2d). Although we did not observe a prominent inflammatory reaction, numbers of epidermal and dermal T-cells, as well as of macrophages, were increased (Supplementary Fig. 2e, f). Control mice in which GFP was activated instead of p16 for the same period did not show these changes (Supplementary Fig. 2g).

Strikingly, proliferation rates in the epidermis of mice expressing p16 for 6 months were dramatically increased, with 81% of basal keratinocytes expressing Ki67 relative to 33% in control mice (Fig. 2f, g). Furthermore, some epidermal regions exhibited dysplasia (atypia), exhibiting changes in nuclear morphology and basal layer organization (Fig. 2f, right panel). Co-staining for p16 and the proliferation marker Mcm7 revealed that 93% of basal IFE keratinocytes in which p16 was not activated (p16^–^) were proliferating, as opposed to 26% of basal IFE cells in control mice (Fig. 2h, i). A corresponding increase in BrdU incorporation, marking cells in S-phase, was observed (Fig. 2j). These findings indicate that prolonged p16 activity in a subset of epidermal cells is sufficient to induce the proliferation of the majority of surrounding keratinocytes. Surprisingly, 30% of p16-expressing (p16^+^) cells also stained positive for Mcm7, and 5% stained positive for BrdU, suggesting that some of the transgene-expressing cells possessed the ability to proliferate (Fig. 2i, j). Chronic epidermal p16 expression thus leads to hyperplasia and regional dysplasia, consistent with paracrine stimulation emanating from the p16^+^ cells.

We tested whether p16-induced hyperplasia influenced the regenerative capacity of the skin following wounding. Healing was delayed in mice in which p16 was activated for 2 weeks prior to wounding, and a similar delay was observed in mice in which p16 was activated for 6 months (Supplementary Fig. 3), indicating that the p16-induced hyperplasia does not lead to enhanced regenerative capacity.

### A subset of cells in human actinic keratosis express p16

The appearance of dysplastic regions in p16-expressing mice was reminiscent of early-stage human premalignant epidermal lesions. To establish whether p16^+^ cells are detected within such lesions, we stained sections of human actinic keratosis (AK) lesions, the common precursors to cutaneous squamous cell carcinoma (cuSCC)^34^. Previous studies reported varying percentages and staining patterns of p16 in these lesions^26–28^. We detected p16 protein in 47 of 72 lesions (65%), collected in three medical centers, with a mean of 11.3% p16^+^ cells per lesion in those that contained such cells (0.8–41.8%, *n* = 47) (Fig. 2k, l). p16^+^ cells were often interspersed among or adjacent to dividing, p16^–^, cells (Fig. 2m, n and Supplementary Fig. 4a). An average of 6% of the cells in AK lesions positively stained for SenTraGor, suggesting the presence of senescent cells in them (Supplementary Fig. 4b, c). Rb protein was also detected in these lesions, excluding Rb deletion or silencing, which would render p16 ineffective (Fig. 2k, right panel). Furthermore, as observed in the p16-induced mice, some p16^+^ cells were proliferating, based on Ki67 expression, yet in lower numbers than the surrounding cells (Fig. 2m, n). These findings are consistent with potential crosstalk between p16^+^ and p16^–^ subpopulations in human AK lesions, as suggested by the mouse model.

### p16 promotes formation of carcinogen-induced papillomas

**Fig. 3:**
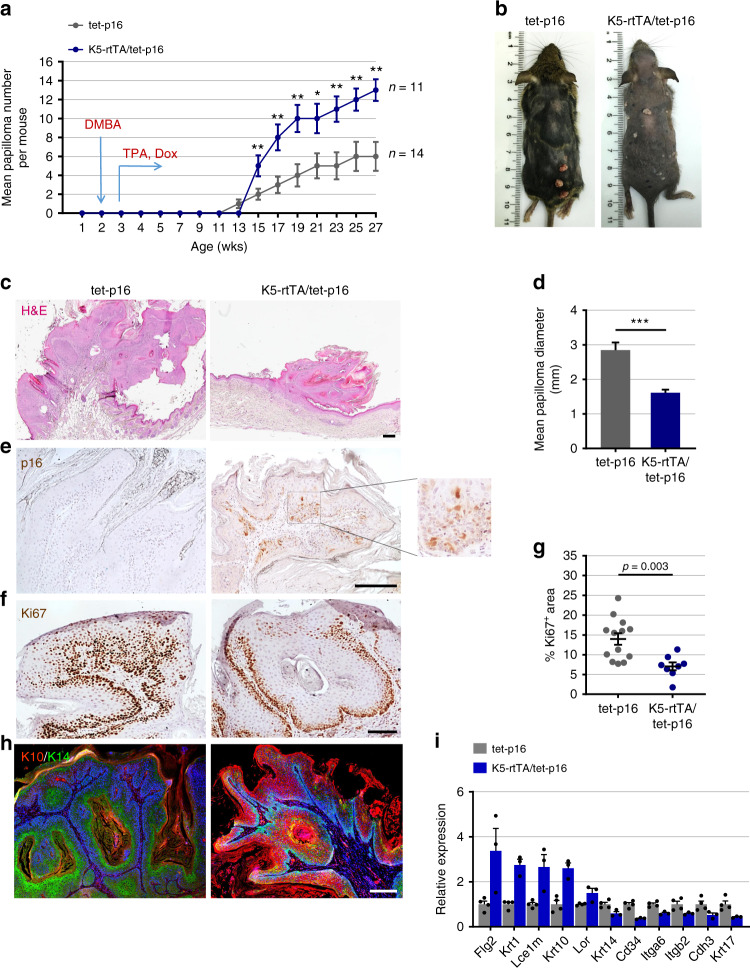
p16 promotes carcinogen-induced papilloma formation but slows lesion growth. **a** Average number of papillomas per mouse formed over time (age in weeks) in K5-rtTA/tet-p16 and sibling control tet-p16 mice treated once with DMBA at 3 weeks, followed by continued TPA treatment and p16 activation by dox starting at 4 weeks. *n* = 14, 11 mice per group combined from four independent experiments. **b** Images of indicated mice at 27 weeks of age, showing papillomas formed on back skins. **c** H&E staining of papillomas from control and p16-induced mice. **d** Mean diameter of papillomas from the same mice at 27 weeks. *n* = 84, 136 papillomas per group. **e** Immunostaining for p16 (brown) in papillomas from same mice. Enlarged inset is shown on right. **f** Staining for Ki67 (brown) in papillomas from same mice. **g** Percentage of Ki67-stained area of the epithelial component of papillomas (dots) from indicated mice, scored by image analysis. *n* = 13,8 in respective groups. **h** Papillomas from control and p16-induced mice stained for K14 (green) and K10 (red), marking the basal and suprabasal layers, respectively. **i** Relative mRNA levels of epidermal differentiation-associated genes in whole papillomas from control and p16-expressing mice, measured by mRNA-seq. *n* = 4, 3. *P* < 0.05 for all genes. Graphs indicate mean values across mice (**a**) or across lesions (**d**, **g**, **i**) ± S.E.M. *P < 0.01, **P < 0.001, ***P < 0.0001, *t* test. Scale bars—200 μm, except **f**—100 μm.

The finding that p16 expression was sufficient to induce epidermal hyperplasia and dysplasia raised the possibility that cells carrying oncogenic mutations would be influenced by a p16-activated environment. We, therefore, tested whether chronic p16 expression would affect the rates of papilloma formation after carcinogen treatment, using the DMBA/TPA model^35^. We treated K5-rtTA/tet-p16 mice, as well as sibling control tet-p16 mice, with a single topical dose of the mutagen DMBA at 3 weeks of age, and 1 week later initiated p16 activation by dox treatment, and simultaneously began twice-weekly topical treatments with TPA. We tracked papilloma formation for 5 months. Strikingly, p16-expressing mice developed approximately double the number of papillomas than control mice (Fig. 3a, b). This indicates that p16 generates an environment that supports the ability of keratinocytes bearing an oncogenic mutation to initiate papillomas.

Interestingly, the papillomas that developed in p16-expressing mice were smaller than those in control mice, and contained fewer proliferating cells, which were more restricted to the basal epidermal regions of the lesions (Fig. 3c–g and Supplementary Fig. 5a). p16^+^ cells were readily detected within these papillomas (Fig. 3e). SenTraGor staining indicated higher numbers of senescent cells within the p16-induced papillomas, relative to the adjacent non-transformed epidermis (Supplementary Fig. 5b,c), consistent with previous reports indicating that p16 exerts a stronger pro-senescent effect when activated in the context of a strong pro-mitotic signal^36^. Expression profiling of whole dissected papillomas revealed a dramatic elevation in markers of differentiated epidermal layers and reduced expression of stem cell-associated markers, in addition to reduced expression of cell cycle, signaling, and cytoskeletal genes (Fig. 3h, i and Supplementary Fig. 5d, e).

Together these findings indicate that p16-expressing cells in the non-transformed tissue stimulate neighboring mutated oncogene-expressing cells to form growing premalignant tumors. However, p16 expression within formed oncogene-expressing lesions restricts proliferation, increasing senescence and differentiation, and thereby reduces tumor growth, consistent with its known tumor-suppressive roles.

### p16 expression activates Wnt in epidermal keratinocytes

**Fig. 4:**
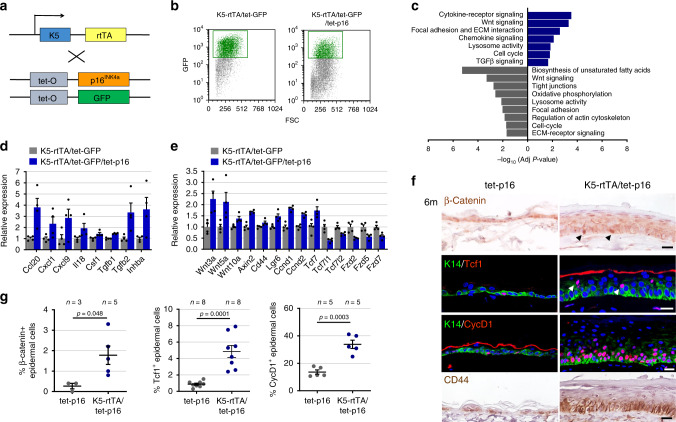
p16 expression activates Wnt-pathway-associated genes. **a** Diagram of transgenic mouse lines crossed for co-induction of GFP and p16. **b** FACS plots showing GFP levels and gates used for isolation of GFP^+^ epidermal cells from K5-rtTA/tet-GFP/tet-p16 (right) and control K5-rtTA/tet-GFP (left) mice, after 6 months of induction. Plots show the CD31^−^/CD140a^−^/CD45^−^ cell fraction only. **c** Gene sets whose expression was preferentially upregulated (blue) or downregulated (grey) in GFP^+^ cells from p16-expressing mice relative to GFP^+^ cells from control mice. Values indicate –log_10_ (adjusted *P* value) by hypergeometric test. **d** mRNA levels of genes encoding cytokines and genes associated with the TGFβ pathway upregulated in p16-expressing GFP^+^ cells, measured by mRNA-seq. Values were normalized to the mean expression levels in GFP^+^ cells from control mice, defined as 1. *P* < 0.05 for all genes, *n* = 4 samples per group, each pooled from 3 mice. **e** Relative mRNA levels of genes associated with the Wnt pathway in the same samples. *P* < 0.05 for all genes, *n* = 4 samples per group, each pooled from 3 mice. **f** Skin sections from K5-rtTA/tet-p16 and control tet-p16 mice treated with dox for 6 months stained for β-Catenin, Tcf1, Cyclin D1 or CD44. Arrowheads indicate representative positively stained cells. **g** Percentages of nuclear β-catenin^+^, Tcf1^+^, and CyclinD1^+^ IFE cells in control and p16-expressing mice after 6 months of induction, as scored visually from images. *P* values calculated by *t* test. All graphs indicate mean across mice ± S.E.M. Scale bars—20 μm.

We next set out to uncover the mechanisms by which p16-expressing cells stimulate proliferation and induce hyperplasia. We generated triple-transgenic K5-rtTA/tet-p16/tet-GFP mice, in which p16 and GFP were co-activated, and treated the mice with dox for 6 months (Fig. 4a). Co-expression of GFP did not reduce the numbers of detected p16^+^ cells (Supplementary Fig. 6a, b). We then isolated GFP^+^ keratinocytes from these mice and from K5-rtTA/tet-GFP control mice expressing only GFP (Fig. 4b and Supplementary Fig. 6c). The transcriptome of GFP^+^ cells from p16-expressing mice revealed changes in multiple genes relative to GFP^+^ cells from control mice (624 upregulated and 750 genes downregulated, Supplementary Data 1), representing pathways known to be involved in senescence, including cell-cycle regulation, cytoskeletal structure, cell adhesion, protein turnover and metabolism (Fig. 4c). Several cytokine-encoding genes were upregulated, including *Ccl20*, *Cxcl1,* and *Cxcl9*, as well as *Tgfβ* ligands, yet these did not constitute a full SASP (Fig. 4d). Interestingly, we found that the levels of several genes encoding Wnt ligands, as well as known Wnt transcriptional target genes, were elevated, while Fzd receptor-encoding genes showed reduced mRNA levels (Fig. 4e). *Tcf7*, which encodes the Wnt-target-activating Tcf1 transcription factor (and a Wnt target itself)^37,38^ was upregulated; conversely, *Tcf7l1* and *Tcf7l2*, which respectively encode the Tcf3 and Tcf4 factors and are known to often act as Wnt target repressors^37,38^, were downregulated, consistent with transcriptional target activation (Fig. 4e). The proliferation-associated *Ccnd* genes (encoding Cyclin D proteins) which also respond to Wnt, were among the upregulated genes, as was often previously observed in senescent cells^39^. Consistent with Wnt-pathway activation, we detected by immunohistology increased numbers of cells expressing nuclear β-catenin in the epidermis of p16-expressing mice, as well as cells expressing Tcf1, Cyclin D1 and CD44 (Fig. 4f, g).

Isolation and transcriptome analysis of the GFP^–^ cell fraction from p16-expressing mice revealed that, relative to the GFP^+^ cells from the same mice, this fraction was enriched for proliferation-associated gene sets (as expected from their hyperproliferative state), and showed reduced expression of senescence-related sets, such as oxidative stress response, protein translation, and cytoskeletal reorganization (Supplementary Fig. 6c–e). Keratinocyte differentiation genes were somewhat elevated, reflecting the presence of diverse differentiation states in the expanded epidermis (Supplementary Fig. 6f). We found that, similar to the GFP^+^ p16-expressing cells, the GFP^–^ fraction showed elevation in Wnt-associated genes relative to cells from control mice, and that the *Tcf7l1* gene was further silenced in these cells (Supplementary Fig. 6g). Together these findings indicate that p16 expression leads to activation of Wnt-pathway genes in both the p16-expressing cells and the surrounding IFE cells. The activation of the Wnt pathway by p16 appeared to occur gradually after prolonged p16 expression, as we did not observe elevation in Wnt-associated genes after 2 days or 2 weeks of p16 induction (Supplementary Fig. 6h).

### p16-induced Wnt secretion drives keratinocyte proliferation

**Fig. 5:**
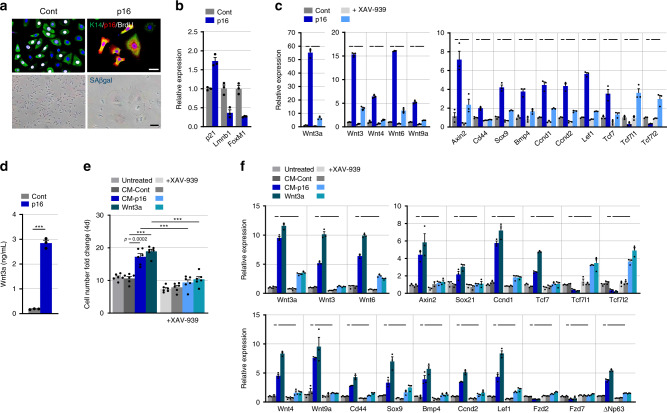
p16-overexpressing primary keratinocytes stimulate proliferation of naive cells through Wnt secretion. **a** Primary mouse keratinocytes infected with a p16-expressing or control lentivirus, co-stained for K14 (green), p16 (red) and BrdU (indicating S-phase, white), or for SA-βGal, 10 days after infection. **b** Relative mRNA levels of the indicated genes in the same keratinocytes, measured by qRT-PCR. *n* = 3 replicates. *P* < 0.05 for all genes. **c** Relative mRNA levels of the indicated Wnt-associated genes measured by qRT-PCR in control and p16-expressing keratinocytes. Values are also shown for the same cells treated with the β-catenin inhibitor XAV-939 (5 μM) for 24 h. *n* = 3 replicates. *P* < 0.05 for all comparisons indicated by lines: p16 versus control (dark blue versus dark grey, short lines), and p16 versus p16+XAV-939 (light blue versus dark blue, long lines). **d** Secreted Wnt3a protein levels in medium of control and p16-expressing cells, measured by ELISA. *n* = 3 replicates. ***P < 0.0001 **e** Fold increase over 4 days in numbers of primary keratinocytes cultured with conditioned media from p16-expressing (CM-p16) or from control (CM-Cont) keratinocytes, or with 10 ng/ml recombinant Wnt3a, in the absence or presence of XAV-939 (darker and lighter columns, respectively). *n* = 6 replicates. ***P < 0.0001. **f** Relative mRNA levels of the indicated Wnt-associated genes measured by qRT-PCR in keratinocytes treated for 24 h with conditioned media from p16-expressing or control cells, or with Wnt3a, in the presence or absence of XAV-939. *n* = 3 replicates. *P* < 0.05 for all comparisons indicated by lines: CM-p16 versus CM-Cont (dark blue versus dark grey, short lines), and CM-p16 versus CM-p16+XAV-939 (light blue versus dark blue, long lines). All graphs indicate mean across replicates ± S.E.M., *t* test. Scale bars—50 μm.

To better dissect p16-induced Wnt activity, we overexpressed p16 in cultured primary mouse keratinocytes by lentiviral transduction. p16 overexpression led to rapid cell-cycle arrest, acquisition of a senescent morphology, and expression of senescence-associated markers, yet, interestingly, with no evidence of SA-βGal activity (Fig. 5a, b). Within 10 days of p16 expression, the levels of multiple Wnt ligands and targets were upregulated in the p16-expressing cells (Fig. 5c). ELISA measurement indicated that Wnt3a levels in conditioned medium (CM) of p16-expressing cells was elevated, reaching 3 ng/ml (Fig. 5d). Treatment of the cells with XAV-939, a Tankyrase1/2 inhibitor that causes β-catenin degradation^40^, suppressed the upregulation of Wnt target genes, including the genes encoding Cyclin D1 and D2, as well as of Wnt ligands (Fig. 5c). The activator *Tcf7* gene was upregulated in p16-expressing cells, whereas the repressors *Tcf7l1* and *Tcf7l2* were both downregulated by p16 overexpression, as was observed in the transgenic mice, and XAV-939 treatment reversed this effect (Fig. 5c). These findings indicate that p16 is sufficient to activate the Wnt pathway in keratinocytes, driving both target and ligand activation in a β-catenin-dependent manner.

To test whether Wnt activation and secretion by p16-expressing keratinocytes can influence naive cells, we treated proliferating primary keratinocytes with conditioned media from p16-expressing or control (empty vector-expressing) keratinocytes, or, as a positive control, with recombinant Wnt3a. While control-cell CM had no effect on the numbers of treated cells, the CM of p16-expressing cells led to enhanced proliferation of the treated cells, resulting in a 1.7-fold increase in cell numbers after 4 days of treatment, similar to the effect of Wnt3a (Fig. 5e). This stimulatory effect was almost completely lost when XAV-939 was administered together with CM or Wnt3a, indicating that proliferation is β-catenin mediated (Fig. 5e). Treated cells showed upregulation of Wnt target genes and downregulation of *Tcf71,2*, as well as increased levels of genes encoding Wnt ligands (Fig. 5f), mirroring the changes observed in the p16-expressing mice. These changes were blocked by XAV-939 (Fig. 5f). Together, these findings indicate that Wnt secretion by p16-expressing cells can stimulate the proliferation of naive keratinocytes, and that Wnt signals act on both the p16-expressing cells themselves and on the non-expressing proliferating cells, activating transcriptional targets as well as Wnt ligands, in a β-catenin-dependent manner (Supplementary Fig. 7).

### The Wnt-pathway mediates p16-induced hyperplasia

**Fig. 6:**
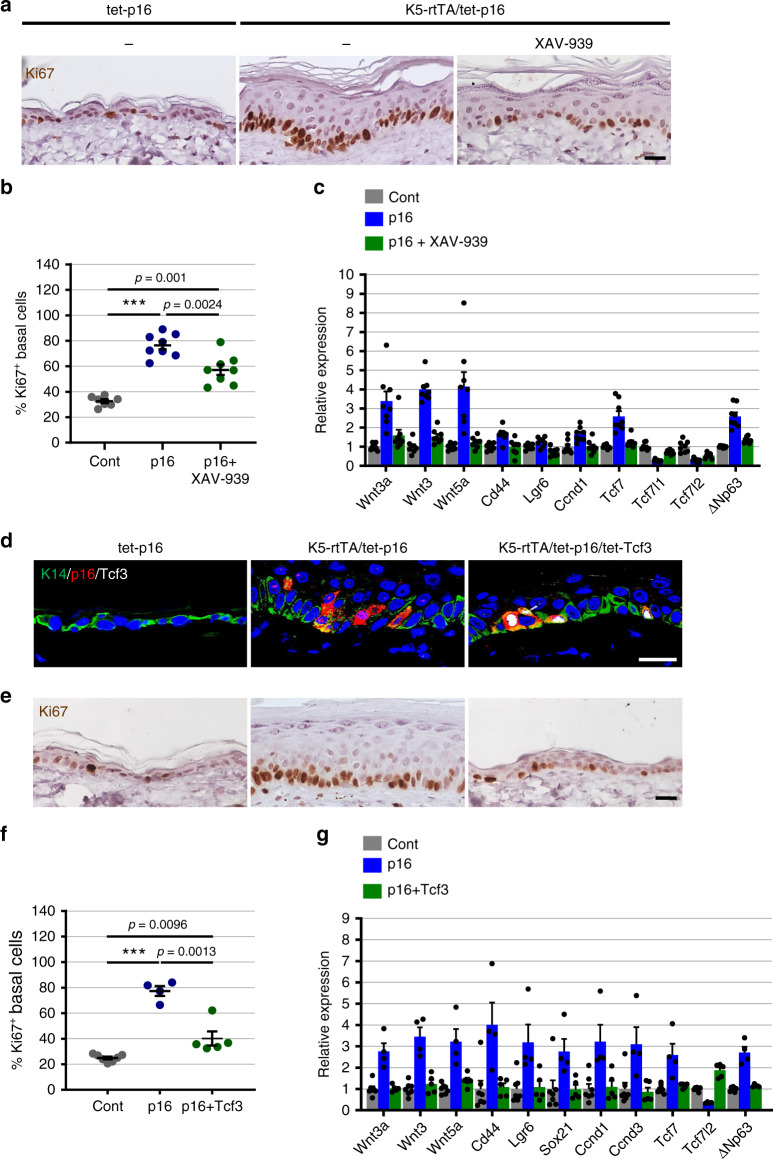
Wnt activity mediates p16-induced epidermal hyperplasia. **a** Skin sections from control tet-p16 mice (left) and K5-rtTA/tet-p16 mice treated with dox for 6 months, and subsequently treated for 1 week with XAV-939 (right) or with vehicle (middle), stained for Ki67 (brown). **b** Percentage of Ki67^+^ basal IFE cells in sections from same mice (dots). *n* = 7, 8, 8, combined from two independent experiments. **c** Relative mRNA levels of the indicated Wnt-associated genes in isolated epidermis from the same mice, measured by qRT-PCR. *n* = 8 mice per group. *P* < 0.05 for all comparisons between p16-expressing mice (blue) and XAV-939-treated mice (green). **d** Skin sections from control (tet-p16), K5-rtTA/tet-p16 and K5-rtTA/tet-p16/tet-Tcf3 mice treated with dox for 6 months, stained for p16 (red) and Tcf3 (white). **e** Skin sections from the same mice as in **d** stained for Ki67 (brown). **f** Percentage of Ki67^+^ basal IFE cells in mice expressing p16 (p16) or p16 together with Tcf3 (p16+Tcf3) for 6 months, and from controls (Cont). *n* = 7, 4, 5. **g** Relative mRNA levels of the indicated Wnt-associated genes in isolated epidermis from the same mice, measured by qRT-PCR. *n* = 7, 4, 5. Scores were done visually from images. All graphs indicate mean values across mice ± S.E.M. *P* < 0.05 for all comparisons between p16-expressing mice (blue) and mice co-expressing p16 and Tcf3 (green). ***P < 0.0001. *t* test was used for all significance tests. Scale bars—20 μm.

We next tested whether Wnt activation contributes to the hyperplasia observed in p16-expressing mice. We activated p16 for 6 months, and then treated the mice systemically with XAV-939 for 1 week. XAV-939 treatment led to a significant reduction in epidermal proliferation rates, as well as a reduction in the expression of Wnt ligand and target genes (Fig. 6a–c). Wnt inhibition was therefore sufficient for a partial reversal of the ongoing hyperplastic effects of p16 expression.

We next tested whether co-activation of the repressive Tcf3 (Tcf7l1) together with p16 would influence Wnt-pathway activation and hyperplasia. We crossed K5-rtTA/tet-p16 mice with mice carrying an inducible Tcf3 transgene (tet-Tcf3)^41^ to generate triple-transgenic mice. Co-activation of Tcf3 together with p16 for 6 months resulted in significantly reduced epidermal hyperplasia, as well as in reduced levels of Wnt ligand and target genes (Fig. 6d–g). Tcf3 expression thus largely blocks the pro-proliferative effects of p16. Together these results indicate that activation of the Wnt pathway upon chronic p16 expression contributes to the induction of epidermal hyperplasia.

### Senolytic elimination of p16^+^ cells suppresses hyperplasia

**Fig. 7:**
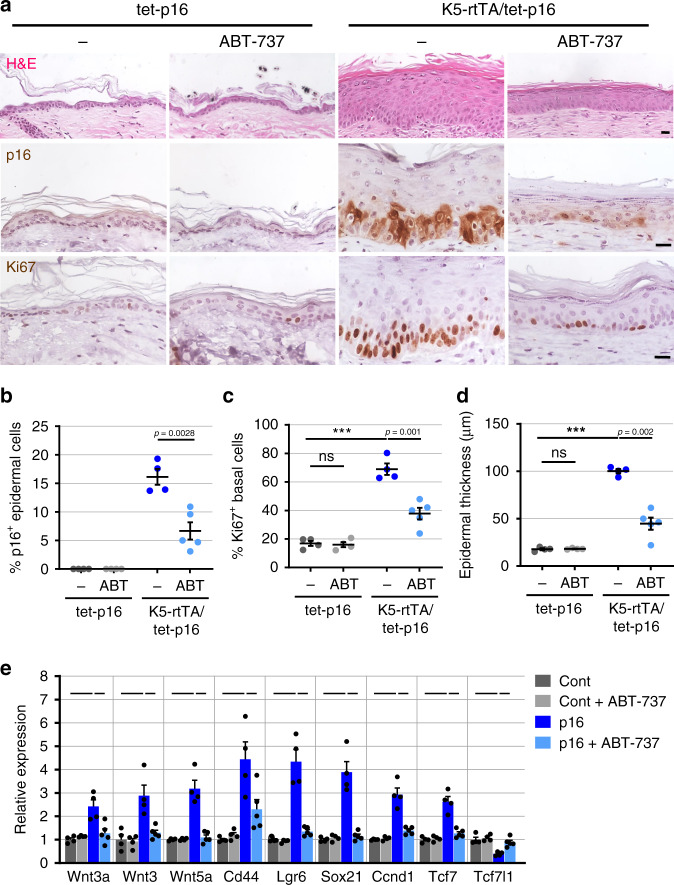
Senolytic treatment inhibits p16-induced hyperplasia and Wnt activation. **a** Skin sections from K5-rtTA/tet-p16 and control tet-p16 mice treated with dox for 6 months and subsequently treated with ABT-737 (6 injections over 9 days), stained by H&E, for p16, or for Ki67. **b** Percentage of p16^+^ IFE cells in ABT-737-treated and untreated p16-induced and control mice (dots). **c** Percentage of Ki67^+^ basal IFE cells in same mice. **d** Epidermal thickness in same mice. **e** Relative mRNA levels of the indicated Wnt-pathway-associated genes in whole skins isolated from the same mice, measured by qRT-PCR. *P* < 0.05 for all comparisons indicated by lines: p16 versus control (dark blue versus dark grey, long lines) and p16+ABT-737 versus p16 (light blue versus dark blue, short lines). *n* = 4, 4, 4, 5 in respective groups in all panels. All graphs indicate mean values across mice ± S.E.M. Scores were conducted visually from images. ***P < 0.0001, *t* test. Scale bars—20 μm.

To study whether the continued presence of p16-expressing cells in the epidermis is required for maintenance of hyperplasia, even after prolonged stimulation, we tested whether these cells can be eliminated by senolytic treatment. The Bcl-2 protein-family inhibitor ABT-737 and its analog ABT-263 (navitoclax) inhibit Bcl-2, Bcl-xl, and Bcl-w, and were shown to have senolytic activity in various settings^16,42,43^. We previously showed that ABT-737 eliminates epidermal cells in which the p53 activator, p14^ARF^, was overexpressed^42^. However, it was unclear, in light of the partial induction of senescence by p16, whether ABT-737 would be effective in eliminating p16-expressing cells. Following six months of p16 induction, we treated mice with ABT-737 six times over nine days and then sacrificed the mice. ABT-737-treated mice showed a significant reduction in the percentage of p16^+^ cells in the IFE (an approximate 60% reduction), and a corresponding reduction in basal cell proliferation and epidermal thickness, indicating partial reversal of the hyperplasia (Fig. 7a–d). Furthermore, the expression of Wnt ligands and targets was significantly reduced in the ABT-737-treated mice (Fig. 7e). p16-expressing cells are thus sensitive to senolytic treatment, and their residence in the epidermis is necessary for the ongoing maintenance of Wnt stimulation and consequent hyperplasia.

## Discussion

The function of p16 as one of the most important tumor suppressor genes is well established, and is supported by its recurrent loss-of-function alterations in a wide variety of human malignancies and by numerous studies in which it was shown to block immortalization and tumorigenesis^4^. An important additional dimension to p16 function is its activation, often linked to cellular senescence, in a subset of cells within aging tissues. Recent studies revealed that p16-expressing cells contribute to aging processes and to associated diseases, and have highlighted the question of whether such resident cells influence the propensity of tissues to develop cancer^11,16^. Our findings reveal that the continuous expression of p16 itself in the epidermis has pro-proliferative and pro-tumorigenic effects. p16 expression, when activated after carcinogen treatment, promoted the initiation of papillomas, indicating that it generates an environment that supports the ability of cells carrying an oncogenic mutation to initiate tumors. p16 may thus function in some settings as a double-edged sword, in the short-term blocking proliferation of damaged cells, but, over time, stimulating the proliferation of neighboring cells and thereby contributing to the formation of premalignant lesions.

Our findings indicate that Wnt signaling plays a central role in mediating p16-induced hyperplasia. Wnt and β-catenin are known to regulate the acquisition of stem cell traits, particularly of bulge hair-follicle stem cells, and also to promote the proliferation of interfollicular epidermal cells and the formation of epidermal tumors^44–47^. p16 overexpression, both in vivo and in cultured primary keratinocytes, led to Wnt ligand secretion and Wnt-pathway activation, stimulating the hyperproliferation of target cells that do not express p16. Interestingly, the recipient cells not only activated Wnt transcriptional targets, but also upregulated Wnt ligand expression, thereby generating a positive feedback loop. In both the p16-expressing and the naive cells, Wnt ligand activation was β-Catenin dependent. This mode of combined autocrine and paracrine stimulation is consistent with previous dissection of Wnt activity in the IFE, in which it was shown that basal cells expressing the Wnt target Axin2 also secrete Wnt ligands, in a β-Catenin-dependent manner^44^. Wnt over-activation in the p16-expressing epidermis occurred gradually over a prolonged period. In contrast, in cultured keratinocytes, Wnt-pathway genes were activated within days of p16 overexpression. The reasons for this difference are unclear, but may be due to more stringent negative feedback loops active in the living tissue.

Wnt activation could be expected to also promote stem cell identity and function in p16-expressing skins. Recent studies have shown that senescence can promote stem cell-like traits in some settings^48–50^. However, we found that p16 expression blocked the proliferation of hair-follicle stem cells, and led to their gradual depletion from the tissue, results similar to those observed in a previous study^51^. Wound healing, a stem cell-dependent process^52^, was delayed in the p16-expressing mice, even during epidermal hyperplasia. It is therefore possible that additional actions of p16 counteract the stimulation of stem cell function by Wnt, particularly in the days immediately following p16 activation, in which the proliferation of HFSCs is arrested and Wnt levels are not yet elevated.

p16 expression induced only partial features of senescence. Numbers of SA-βGal^+^ and SenTraGor^+^ cells were elevated, but were lower than those of p16^+^ cells. We observed an increase in keratinocyte size, a hallmark of senescence, and changes in senescence-associated gene sets, such as genes involved in cytoskeletal structure and protein translation. Yet various other known markers of senescence^53^ were unchanged. Several cytokine-encoding genes were upregulated in p16-expressing cells, and while these could potentially contribute to the observed phenotypes, they did not constitute a full SASP response, which typically involves high levels of multiple cytokines. This is consistent with previous observations that p16 does not activate the SASP^20^. While several past studies have linked Wnt ligand activation to senescence, the paracrine actions of Wnt were not addressed, and in some settings Wnt activity was reported to counteract senescence^50,54–56^. Our work thus reveals a tumor-promoting contribution of p16 that does not necessitate a broad SASP but acts mainly through Wnt.

Accumulation of p16-expressing cells may preferentially occur in epidermal regions exposed to damage. p16 is known to be activated by UVR, the major cause of human skin malignancies^13,25^. UV-irradiated mouse skins show a thickening reminiscent of that induced by p16 overexpression^57^. Expanding previous observations^26–28^, we show that p16 is indeed often expressed in actinic keratosis lesions, which are among the most common human premalignant growths and are induced by UVR. Furthermore, we show that p16^+^ cells in these lesions are in close proximity to p16^–^ dividing cells, in a pattern similar to that observed in our mouse model. This pattern is consistent with paracrine interaction between these cell subsets, yet such crosstalk between them remains to be directly proven.

Mutations in p53 occur very early in skin tumorigenesis, as a direct consequence of UVR^34^. Interestingly, this may result in further enhanced compensatory p16 expression and senescence in such lesions^9,58^, possibly providing a proliferation-promoting environment. Upon progression to cuSCC p16 is often mutated^29,30^, suggesting that its counter-proliferative functions outweigh the potential benefit of its activity. Our observation that the expression of p16 restricts proliferation within growing papillomas suggests that its activity following tumor initiation blocks further growth, as is expected from its tumor-suppressive function (Supplementary Fig. 7). Interestingly, the major effect of p16 within the papillomas was restriction of proliferation and maintenance of a differentiated state, together with increased senescence. Unlike the untransformed tissue, p16-expressing papillomas did not show overall increased Wnt activation. The actions of p16 in cells that express an active oncogenic driver may thus be distinct from its effects in normal cells.

Pharmacologic elimination of senescent cells through drugs with senolytic activity has been shown in recent studies to be beneficial in a variety of preclinical disease models, and some of these drugs are currently being tested in clinical trials^16,17,59^. Our findings suggest that senolytic treatment may be beneficial for early premalignant epidermal lesions.

Altogether, we reveal in this study a novel dimension of the functions of p16 in skin tumorigenesis, suggesting a non-cell-autonomous contribution to the formation of premalignant lesions, and a potential avenue for blocking this function through senolytic treatment.

## Methods

### Mice

The following transgenic mouse lines were crossed as indicated: tet-p16 (mixed C57Bl6 and 129sv), K5-rtTA (FVB), tet-GFP (mixed C57Bl6 and 129sv) and tet-Tcf3 (FVB). For transgene induction mice received 2 mg/mL doxycycline (Bio Basic) in their drinking water starting at the age of 3 weeks, and treatment was maintained for the indicated durations. In all experiments, the experimental and control mouse groups were siblings grown and treated together. For short-term inductions (up to 2 weeks), both males and females were used; for inductions of 6 months and wound-healing assays all presented results are from male mice, whereas for papilloma formation assays only data of females is shown, due to higher average lesion formation rates. For bromodeoxyuridine (BrdU) labeling mice were injected intraperitoneally with 100 mg/Kg BrdU 2 h prior to sacrifice. For Wnt-pathway inhibition, mice that received dox for 6 months were injected intraperitoneally with 200 μg XAV-939 (Sigma-Aldrich) in 10% DMSO/90% 0.9% NaCl twice a day for seven consecutive days. ABT-737 (WuXi AppTec), was prepared at 75 mg/kg in 30% propylene glycol, 5% Tween 80, 3.3% dextrose in water pH 4–5, and mice were injected intraperitoneally 6 times over 9 days after 6 months of induction. Control mice were injected with vehicle solutions. Upon sacrifice, mice were shaved and their dorsal skins were paraffin-embedded for immunohistology or frozen in OCT solution for SA-βgal staining. Chemically-induced skin carcinogenesis was conducted as described^35^. Three-week-old K5-rtTA/tet-p16 and control tet-p16 mice were shaved, and one day later received a single topical treatment of 50 nmole DMBA (7,12-Dimethylbenz[*a*]anthracene) in 200 μl acetone. After one week dox was added to the drinking water and the mice began twice-weekly topical treatments with 4 μg TPA (12-O-tetradecanoyl phorbol-13-acetate) in 200 μl acetone. Mice were examined for 23 additional weeks for papilloma detection and measurement and were sacrificed 10 days after the last TPA treatment. Papilloma numbers were scored weekly, with diameters measured by caliper. Only papillomas with ≥1 mm diameter were scored. Individual whole papillomas were excised and processed for RNA extraction and profiling, or paraffin embedded. For wound-healing assays dorsal skins were shaved and treated with depilating cream two days prior to wound formation; mice were anesthetized, their back skin was sterilized with povidone-iodine solution, and 1 cm^2^ full-thickness excision wounds were introduced to the upper paravertebral area using sterile scissors. Wounds were measured daily with a caliper. Animals were housed in specific pathogen-free conditions, at 20–24 °C, 30–70% humidity and a 12:12 h light-dark cycle. The Hebrew University is an AAALAC International accredited institute. All experiments were conducted with approval from the Hebrew University Animal Care and Use Committee.

### Cultured keratinocytes

Epidermal keratinocytes were isolated following published procedures^60^. Dorsal skin was removed from newborn mice, and was incubated with dispase (Sigma-Aldrich) followed by isolation of the epidermis and treatment with trypsin (Biological Industries). Keratinocytes were plated on fibroblast feeder cells for four passages and then plated in regular tissue culture dishes. Passage 16 cells were infected with a lentivirus expressing human p16 (pLV-hCDKN2a-GFP-Puro, Vector Builder) or a control empty lentiviral vector, and selected with puromycin (2 μg/mL). 10 days after infection cells were fixed and stained as indicated, or lysed for RNA extraction. Conditioned medium (CM) was collected from cells after 3 days of culture and filtered through a 0.45 μm filter. Wnt3a levels in CM were measured using Wnt3a ELISA kit (R&D Systems, DY1324B-05). Naive keratinocytes were treated with CM in a 1:1 ratio with fresh media, or with 10 ng/ml recombinant Wnt3a (R&D Systems). RNA was collected from treated cells one day after treatment. For proliferation measurements, 10^4^ naive keratinocytes were plated in 24-well plates, treated with CM from p16-expressing or control cells, or with Wnt3a (6 replicates for each), and cell numbers were scored after 4 days. XAV-939 (Sigma-Aldrich, 5 μM) was added where indicated and control samples were treated with DMSO vehicle.

### Human actinic keratosis samples

A total of 72 human actinic keratosis lesions, surgically resected, were collected in three medical centers: 19 patient samples were obtained from the laboratory of Histology-Embryology, Athens Medical School under approval of the Bio-Ethics Committee of Athens Medical School, in accordance with the Declaration of Helsinki and local laws and regulations, following written consent from patients; 42 lesion samples from 35 patients were obtained from the University of Arizona, USA, approved by the University of Arizona Institutional Review Board protocol, with patient informed consent; 11 patient samples were obtained from the Hadassah-Hebrew University Medical Center, Jerusalem, Israel under approval of the Hadassah Medical Center Helsinki Committee for usage of fully anonymized archival specimens, and approved waiver of patient consent. All slides underwent pathologist review to verify lesion type. Patients were aged 38 to 92, with equal representation of males and females. All available patient samples were included in the analysis, with no selection.

### Immunohistology and immunocytology

Immunohistologic stains were performed on 5 μm paraffin sections of collected mouse back skins, individual papillomas, or human AKs, according to standard protocols, using Peroxidase Substrate Kit (Vector) or fluorescently-labeled secondary antibodies (Jackson). The following primary antibodies were used: human p16^INK4a^ (Abcam, ab108349), human p16^INK4a^ (BD Pharmingen, 551153), p-Rb (Santa Cruz, sc-16669-R), Ki67 (Abcam, ab16667), BrdU (Bio-Rad, MCA2060), Mcm7 (Santa Cruz, sc-9966), E-Cadherin (BD Pharmingen, 610182), K14 (Progen, GP-CK14), K10 (Santa Cruz, sc-23877), K5 (Progen, GP-CK5), Rb (Santa Cruz, sc-102), CyclinD1 (Abcam, ab16663), β-catenin (BD Pharmingen, 610153), Tcf1 (Invitrogen, MA5-14965), Tcf3 (Santa Cruz, sc-8635), CD44 (eBioscience, 14-0441-82), Vimentin (Cell Signaling, 5741), CD3 (Bio-Rad, MCA1477), and F4/80 (Bio-Rad, MCA497). Masson’s Trichrome stain was conducted using staining kit (Sigma-Aldrich, HT15) according to manufacturer instructions. SenTraGor stain was conducted as described^32^. For SA-βGal stains, 12 μm frozen sections of OCT-embedded mouse skins, or cultured keratinocytes, were fixed in 0.5% glutaraldehyde for 15 min and stained using standard procedures^33^. For co-stains of cultured keratinocytes for BrdU, p16, and K14, cells were treated for 2 h with 10 μg/mL BrdU and then fixed with cold ethanol, treated with 1 M HCl followed by 0.1 M sodium tetraborate (Na_2_B_4_O_7_) for neutralization, and then stained, as indicated, using PBS + 10% fetal calf serum (FCS) for antibody suspensions. Brightfield images were collected using an Olympus CX41 and DS-Fi1 camera and processed using NIS-Elements software (Nikon). Fluorescent images were collected using an Olympus FV1000 confocal microscope and processed with the FV10-ASW software. All mouse skin images shown are of back skin. Percentages of SA-βGal^+^, SenTraGor^+^, p16^+^, Ki67^+^, Mcm7^+^, BrdU^+^, CycD1^+^, nuclear β-Catenin^+^, Tcf1^+^ and epidermal CD3^+^ cells, out of total IFE or basal IFE cells as indicated, were visually scored from 5 or more microscopic field images per mouse, and 2 or more fields from human AK lesions, with 100–1000 total cells scored per sample. Absolute numbers per microscopic fields of hair follicles, IFE cells, dermal CD3^+^ and dermal F4/80^+^ cells were scored from 8 or more fields per mouse (10× magnification for HFs, 40× for others). Percentage of Ki67^+^ area in papillomas out of epithelial cell area was measured using NIS-elements software (Nikon). Epidermal thickness was measured by NIS elements on 36 or more measurements per mouse. Keratinocyte area calculation was performed on skin sections co-stained for E-Cadherin and p16 by measuring cell circumference using NIS-Elements, with 60 cells or more measured per mouse.

### FACS analysis

Adult epidermal cells were isolated following published procedures^61^. Mouse back skins were shaved and excised, and adipose and muscle tissues were removed. The skin was then incubated with 0.25% trypsin solution overnight at 4 °C, and the epidermis was scraped off, minced and suspended in a trypsin/PBS with 0.5% BSA solution, followed by filtration through a 70 μm cell strainer. Antibody stains were performed using standard procedures. For hair-follicle stem cell analysis live cells were stained for Sca-1, CD34, and CD49f, with three mice pooled in each group. Isolation of GFP^+^ keratinocytes for mRNA-seq profiling was conducted as described above, and live cells were stained for CD140a, CD45 and CD31 to gate out fibroblasts, immune and endothelial cells, respectively. For scoring of K10^+^ epidermal cell percentage, adult epidermal cells were isolated from ear skin samples to obtain improved dissociation. Ear epidermal layers were obtained by digestion with 2 mg/mL Dispase II (Roche Diagnostics) in PBS solution for 40 min at 37 °C, and treated with 2 mg/mL collagenase type II (Worthington Biochemicals) and 1 mg/mL DNase I (Roche) in PBS with 2% fetal calf serum for 25 min at 37 °C. Samples were then incubated with 0.5 M EDTA for 10 min and filtered through a 70 μm strainer. Cells were treated with fixation and permeabilization solution (BD Pharmingen) for 15 min and stained for K10 and secondary fluorescent-conjugated antibody. Samples were sorted on a FACS Aria III system using FACSDiva software (BD Biosciences), or were analyzed on a MACSQuant Analyzer (Miltenyi Biotec). Post-acquisition analysis was conducted with FCS Express (De Novo Software). Antibodies used: CD34 (BD Bioscience, 562608), Sca-1 (Biolegend, 108113), CD49f (eBioscience, 17-0495-80), K10 (Santa Cruz, sc-23877), CD45 (eBioscience, 25-0451-82), CD140a (eBioscience, 17-1401-81), and CD31 (eBioscience, 17-0311-80).

### RNA extraction, qRT-PCR, and expression profiling

Total RNA from whole skin samples and whole papillomas was extracted using the Tissue RNA purification kit (Norgen Biotek). RNA from isolated epidermis was extracted following epidermal separation using the RNeasy Minikit (Qiagen). RNA extraction from FACS-sorted cells was done using the Single Cell RNA Purification Kit (Norgen Biotek). RNA was extracted from primary keratinocytes using Direct-zol kit (Zymo Research, #R2051). cDNA was prepared using iScript cDNA synthesis kit (Bio-Rad), and qRT-PCR was conducted using iTaq Universal SYBR Green Supermix (Bio-Rad) in triplicates, with expression values normalized to *Hprt* and *Ppia* expression levels for mouse and primary keratinocyte samples, respectively. Primer lists are provided in Supplementary Table 1. All RT-PCR graphs conducted on mouse samples indicate values across individual mice. We conducted two mRNA-seq profilings of isolated keratinocytes. In the first, we analyzed 80,000 GFP^+^ keratinocytes from K5-rtTA/tet-p16/tet-GFP and control K5-rtTA/tet-GFP mice, after exclusion of CD45^+^, CD31^+^ and CD140a^+^ cells. Cells were pooled from three mice in each replicate, with four replicates used for each group, obtained in three independent experiments. In the second experiment, we similarly isolated 80,000 GFP^+^ cells from the same mouse groups (after CD45^+^, CD31^+^ and CD140a^+^ exclusion), as well as 20,000 GFP^−^ cells from K5-rtTA/tet-p16/tet-GFP mice, pooling 3 mice per each sample. *n* = 4, 4, 5 for these groups, respectively. mRNA-Seq transcriptional profiling was conducted using an adapted CEL-Seq2 protocol^62^: 3′ cDNA was synthesized and barcoded, followed by RNA synthesis, amplification by in vitro transcription, and library generation for paired-end sequencing. Reads were demultiplexed, quality filtered, and trimmed for adapters and poly-A tail using Cutadapt and aligned with the mouse genome (GRCm38) using Tophat2. Differentially expressed genes were determined by DESeq2, using a *P* < 0.05 cutoff. Quality control and principal component analyses were conducted using R: packages ‘RColorBrewer_1.1-2’, ‘pheatmap_1.0.10’, ‘ggplot2_3.1.0’ and ggrepel_0.8.0. Enrichment of gene sets was determined using the hypergeometric test through Metascape^63^, with adjusted *P* values calculated using the Benjamini-Hochberg procedure. Profiling of papillomas was done with the same method, using RNA from whole isolated lesions, 4 control and 3 p16-expressing. Analysis of gene sets in these comparisons was done for up- or downregulated genes with Log_2_fold change > 0.6 at *P* < 0.05. We excluded in this analysis genes not expressed in keratinocytes, based on our profiling of FACS-sorted populations, to reduce biases introduced by differential inclusion of non-epithelial cell types within the lesions. Gene sets representing epidermal keratinocyte differentiation states were compiled from Joost et al.^64^.

### Statistical analysis

Two-tailed unpaired Student’s *t* test was used to derive *P* values in all comparisons stating usage of *t* test. Other methods were used as indicated.

### Reporting summary

Further information on research design is available in the Nature Research Reporting Summary linked to this article.

## Supplementary information

Supplementary Information

Description of Additional Supplementary Files

Supplementary Data 1

Reporting Summary

## Data Availability

The mRNA-seq expression profiles were deposited in GEO database, under accession number GSE146979 [https://www.ncbi.nlm.nih.gov/geo/query/acc.cgi]. All the other data supporting the findings of this study are available within the article and its supplementary information files and from the corresponding author upon reasonable request. A reporting summary for this article is available as a Supplementary Information file.
