# Supplementary material for: Chronic expression of p16INK4a in the epidermis induces Wnt-mediated hyperplasia and promotes tumor initiation
Source: Nat Commun. 2020 Jun 1;11:2711. doi: 10.1038/s41467-020-16475-3 (PMC7264228; doi:10.1038/s41467-020-16475-3)
Supplement: Supplementary file 1 — Supplementary Information [file 41467_2020_16475_MOESM1_ESM.pdf]

Chronic expression of p16^{INK4a} in the epidermis induces Wnt-mediated hyperplasia and promotes tumor initiation

Azazmeh et al.

Supplementary Information

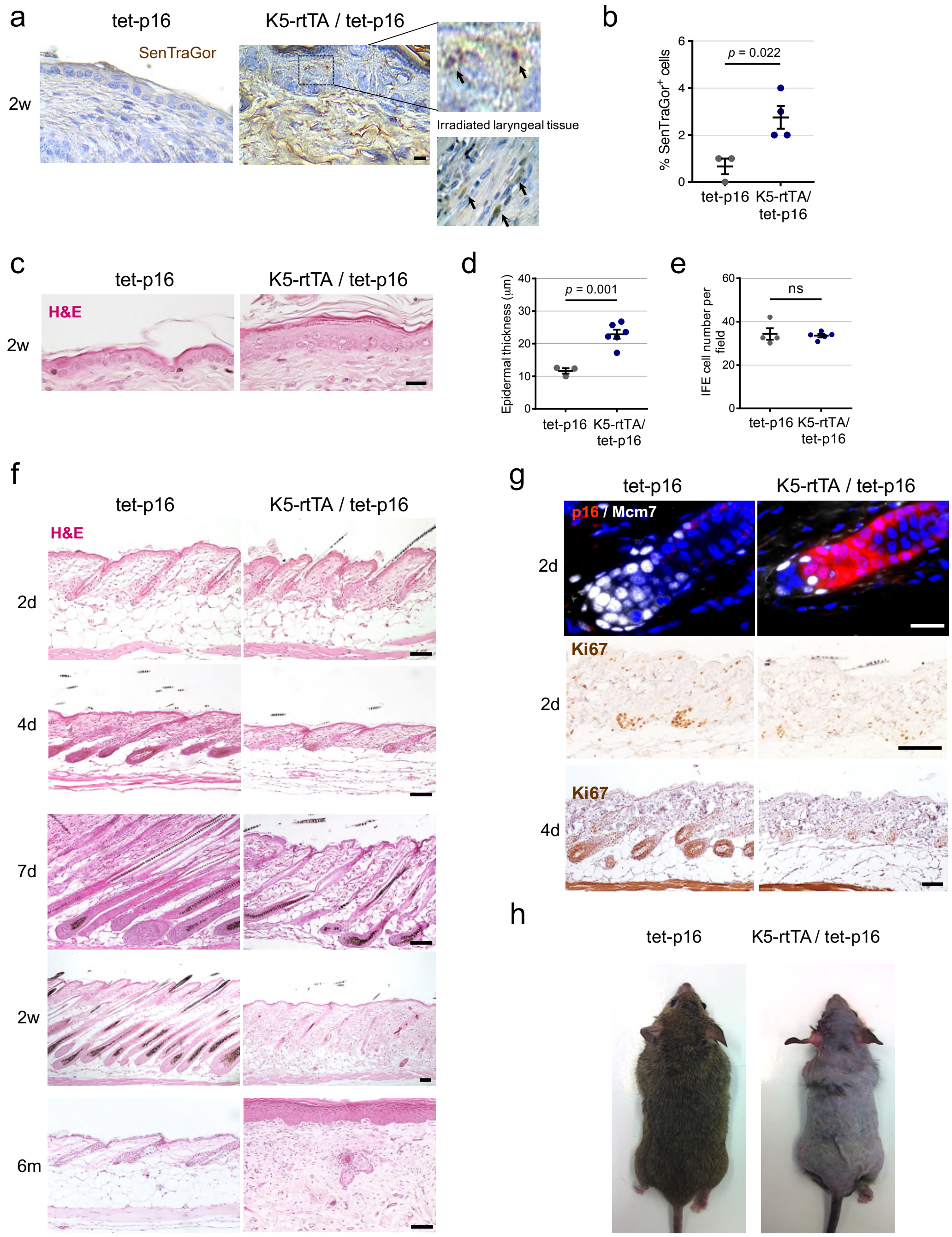

Supplementary Figure 1. p16 induces senescence, cell growth and hair follicle stem cell dysfunction. a) Skin sections from control (tet-p16) and K5-rtTA/tet-p16 mice treated with dox for 2 weeks stained for the SenTraGor senescence marker. Enlarged inset is shown on right (top). Positive cells stain dark brown (arrows). Irradiated laryngeal carcinoma tissue, known to contain senescent cells, is shown as a positive control for the stain on right (bottom). **b)** Percentage of SenTraGor+ IFE cells in the same mice, scored visually from images. n=3,4 for control and p16-expressing mice respectively. **c)** H&E-stained skin sections from indicated mice treated with dox for 2 weeks, showing increased thickness of p16-expressing epidermis. **d)** Epidermal layer thickness of control and p16-expressing mice (dots) after 2 weeks of induction, measured from images. n=3,6. **e)** IFE keratinocyte number per microscopic field (40x) in sections from same mice (dots), scored from images. n=4,5. **f)** Skin sections from mice expressing p16 for the indicated timepoints, all starting at 3 weeks of age, stained by H&E. A delay in hair follicle entry into anagen is observed at 4-7 days of induction, with aberrant follicle development at 7 days and 2 weeks, and follicle loss by 6 months of induction. Images are representative of >5 skin regions in >3 mice per group and were replicated two times or more for each time-point. **g)** Hair follicles from control and mice expressing p16 for the indicated times, co-stained for p16 (red) and the proliferation marker Mcm7 (white), or for the proliferation marker Ki67 (brown). Reduced proliferation of hair follicle cells is observed in p16-expressing skins during anagen. Images are representative of >15 follicles in >3 mice per group and were replicated two times or more for each time point. **h)** Dorsal view of control and mice expressing p16 for 6 months. *t* test. ns – non-significant. Scale bars – 100μm except for panels a, c and g (top) – 20μm.

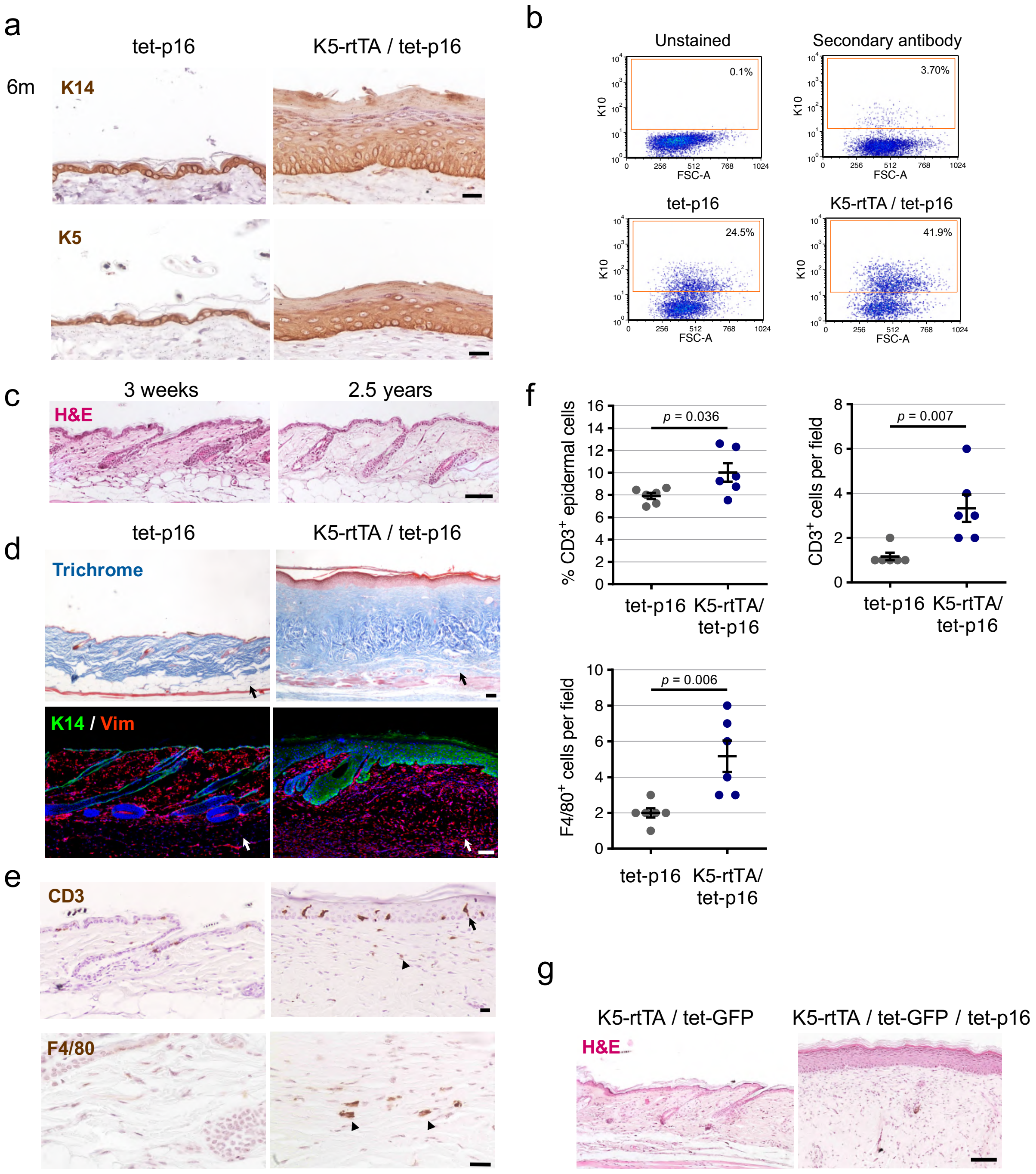

Supplementary Figure 2. Skin histology of p16-expressing mice. a) Skin sections from control tet-p16 and K5-rtTA/tet-p16 mice treated with dox for 6 months stained for the basal markers K14 and K5 (brown), indicating preservation of basal layer identity. Images are representative of >5 skin regions in >3 mice per group in >3 independent experiments. **b)** FACS analysis of K10 expression in epidermal cells isolated from mouse ears of control tet-p16 and K5-rtTA/tet-p16 treated with dox for 6 months. Gates indicate K10⁺ population, top panels show control unstained and secondary stained samples. Quantification of full mouse cohort is shown in Fig. 2e. **c)** H&E-stained skin sections from 3 week- and 2.5-year-old wild type mice. Images are representative of >5 skin regions in 2 mice per group. **d)** skin sections from control tet-p16 and K5-rtTA/tet-p16 mice treated with dox for 6 months, stained by Masson's Trichrome for collagen detection (blue, top panels) or Vimentin (red) (bottom). Arrows indicate regions in which subcutaneous fat was replaced by fibroblasts and collagen. Images are representative of >5 skin regions in >3 mice per group in one experiment. **e)** Sections from same mice stained for the T cell marker CD3 or for the macrophage marker F4/80. Arrow indicates epidermal T-cells, arrowheads indicate dermal T-cells (top) or macrophages (bottom). **f)** Top left – Percentage of $CD3^+$ cells located in the epidermis out of IFE cells, in the same mice (dots), scored visually from images; top right – number of $CD3^+$ cells located in the dermis per microscopic field; bottom – number of dermal $F4/80^+$ cells per microscopic field in the same mice. n=6 per group. **g)** H&E-stained skin sections from K5-rtTA/tet-GFP mice (expressing GFP only) and K5-rtTA/tet-GFP/tet-p16 mice (co-expressing GFP and p16) treated with dox for 6 months. Graphs indicate mean across mice ± S.E.M, all scores were conducted visually from images. *t* test. Scale bars – 20μm for panels a,e, 100μm for panels c,d,g.

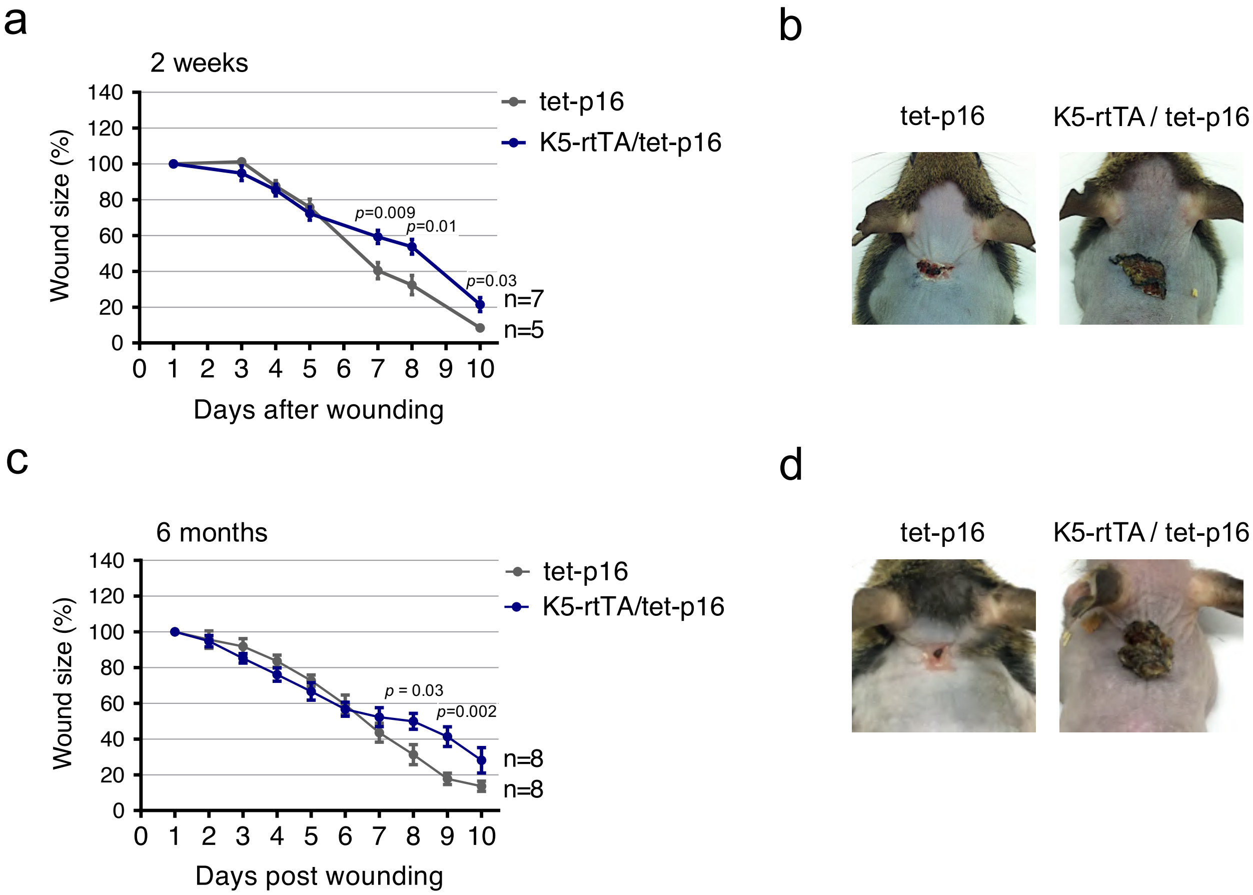

Supplementary Figure 3. p16 delays cutaneous wound healing. a) Wound sizes in days after skin wounding of K5-rtTA/tet-p16 and control tet-p16 mice treated with dox for 2 weeks. Values indicate percentages relative to wound size at day 1. n=5,7 in respective groups. **b)** Representative wounds 8 days after injury in the same mice. **c)** Wound size percentages in days after skin wounding of K5-rtTA/tet-p16 and control tet-p16 mice treated with dox for 6 months. n=8 per group. **d)** Representative wounds 9 days after injury in the same mice. Graphs indicate mean across mice ± S.E.M. *t* test.

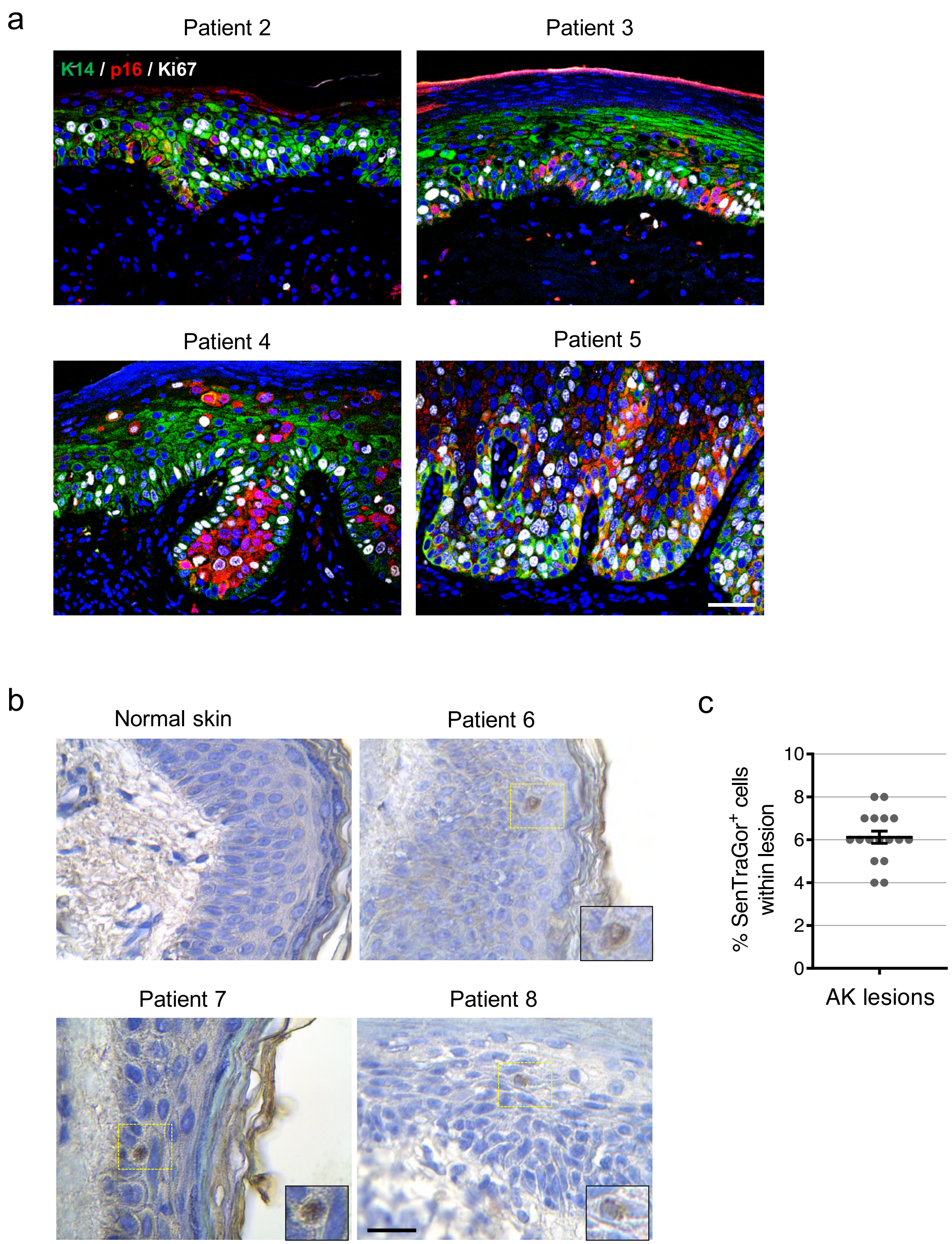

Supplementary Figure 4. p16-expressing cells and senescence in human actinic keratosis lesions. a) Sections of actinic keratosis lesions from four different human patients, stained for p16 (red), Ki67 (white) and K14 (green). Images are additional to the samples shown in Fig. 2m, and representative of the samples quantified in Fig. 2n. **b)** SenTraGor stain of human AKs and a normal skin section (dark brown). Positive staining cells are enlarged in insets. **c)** Percentage of SenTraGor-positive cells in human AK lesions from different patients. n=17. The graph indicates mean across lesions ± S.E.M. Images in panel b show partial area of lesions, SenTraGor^{+} cells were scored throughout lesion area. Scale bars – 50μm.

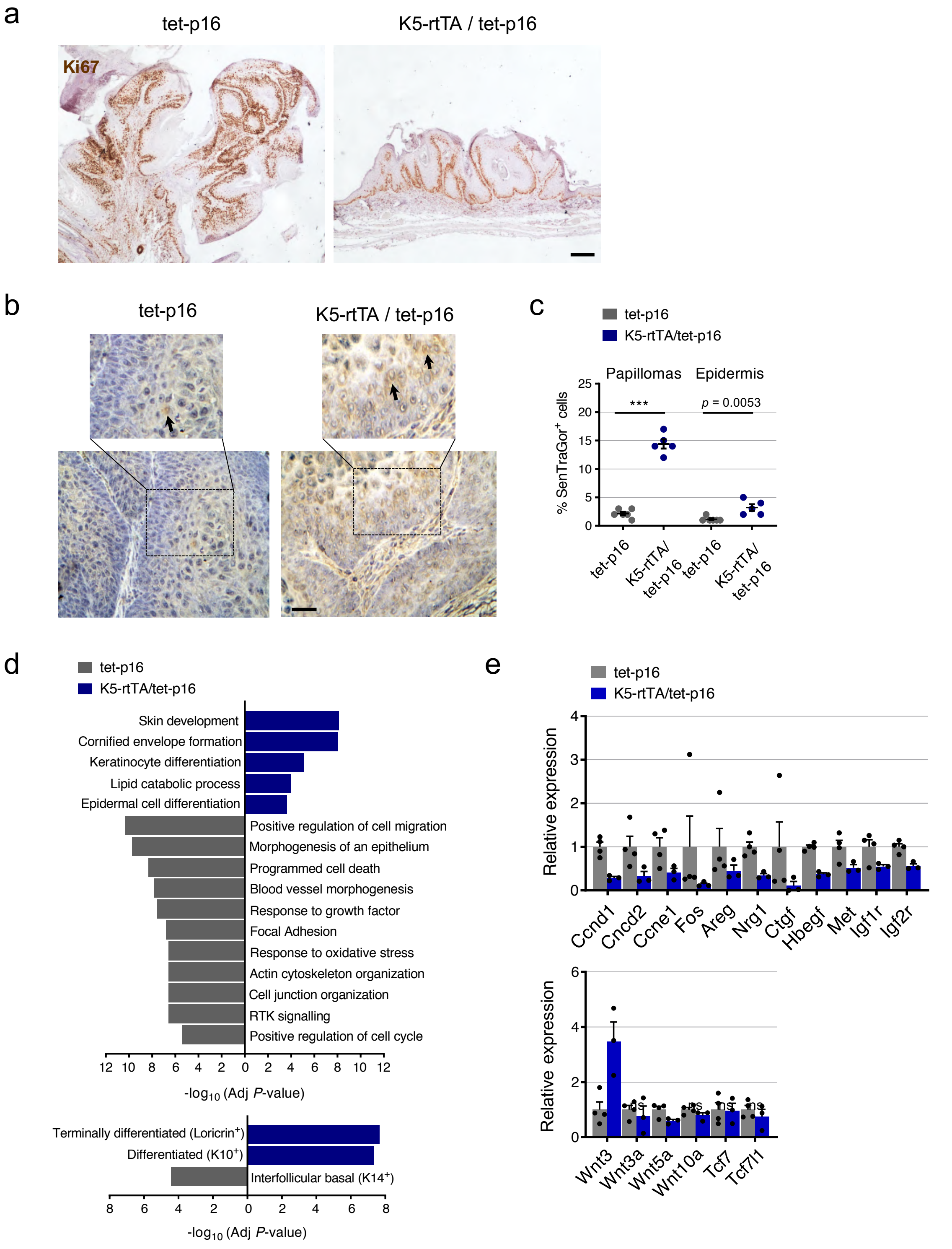

Supplementary Figure 5. p16 suppresses proliferation and induces senescence and differentiation in growing papillomas. a) Low magnification images of papillomas developed in control tet-p16 or K5-rtTA/tet-p16 mice stained for Ki67 (brown), shown in Fig. 3f and quantified in Fig. 3g. **b)** SenTraGor staining (dark brown) of papillomas from the same mice. Arrows indicate positive cells in enlarged insets. **c)** Percentage of SenTraGor positive cells in the epithelial component of papillomas (dots) from the same mice (left panel) and the adjacent IFE (right panel). n= 6,5 in tet-p16 and K5-rtTA/tet-p16 mice, respectively. **d)** Gene sets whose expression was preferentially upregulated in p16-expressing (blue) or control (grey) papillomas, relative to each other. Values indicate $-\log_{10}$ (Adj *P* value) by hypergeometric test. **e)** Relative mRNA levels of the indicated genes in the same samples, measured by mRNA-Seq. n=4,3. All graphs indicate mean across mouse lesions ± S.E.M. *** $P < 0.0001$, ns – non-significant. *t* test. Scale bars – 200µm for panel a, 50µm for panel b.

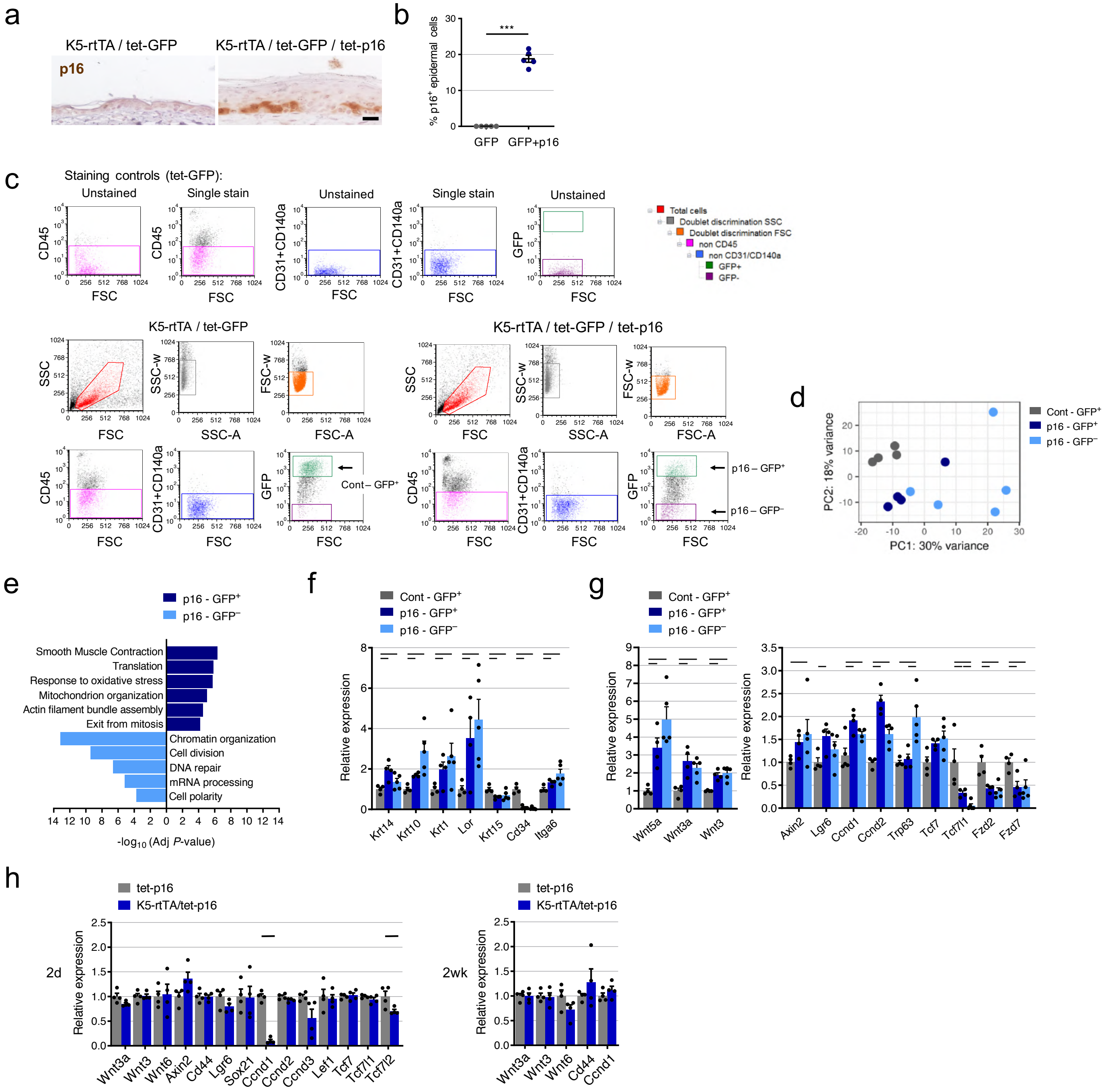

Supplementary Figure 6. Isolation and analysis of keratinocytes from p16-expressing mice. a) Skin sections from K5-rtTA/tet-GFP/tet-p16 (co-expressing p16 and GFP) and control K5-rtTA/tet-GFP mice (expressing GFP only) treated with doxycycline (dox) for 6 months, stained for p16 (brown). **b)** Percentage of p16+ IFE cells in mice expressing GFP, or GFP together with p16. n=5 per group. **c)** FACS isolation of GFP+ and GFP− keratinocytes. Top panels show gating scheme and unstained and single-stained controls, conducted on control mice (single transgene tet-GFP) not expressing neither GFP nor p16. Bottom panels show representative analyses of control K5-rtTA/tet-GFP (left) and p16 expressing K5-rtTA/tet-GFP/tet-p16 mice. CD45− and CD31− CD140a− cells were gated to analyze GFP expression. Arrows indicate three subpopulations collected for mRNA-Seq. **d)** PCA plots of isolated and profiled cell subpopulations. n=4,4,5 in respective groups, each pooled from 3 mice. **e)** Gene sets whose expression was preferentially upregulated in GFP+ cells from p16-expressing mice (dark blue) or in GFP− cells from the same mice (light blue), relative to each other. Values indicate $-\log_{10}$ (Adj *P* value) by hypergeometric test. **f)** Relative mRNA levels of genes encoding differentiation markers in the same samples, measured by mRNA-seq, normalized to levels in Cont GFP+ cells. **g)** Relative mRNA levels of genes encoding Wnt ligands (left) and genes associated with the Wnt pathway (right) in the same samples. **h)** Relative mRNA levels of Wnt-associated genes after 2 days (left) or 2 weeks (right) of p16 induction, as measured by qRT-PCR on mRNA extracted from unsorted epidermis. $P < 0.05$ for all comparisons indicated by lines in panel f,g,h., *t* test. Scale bar – 20μm.

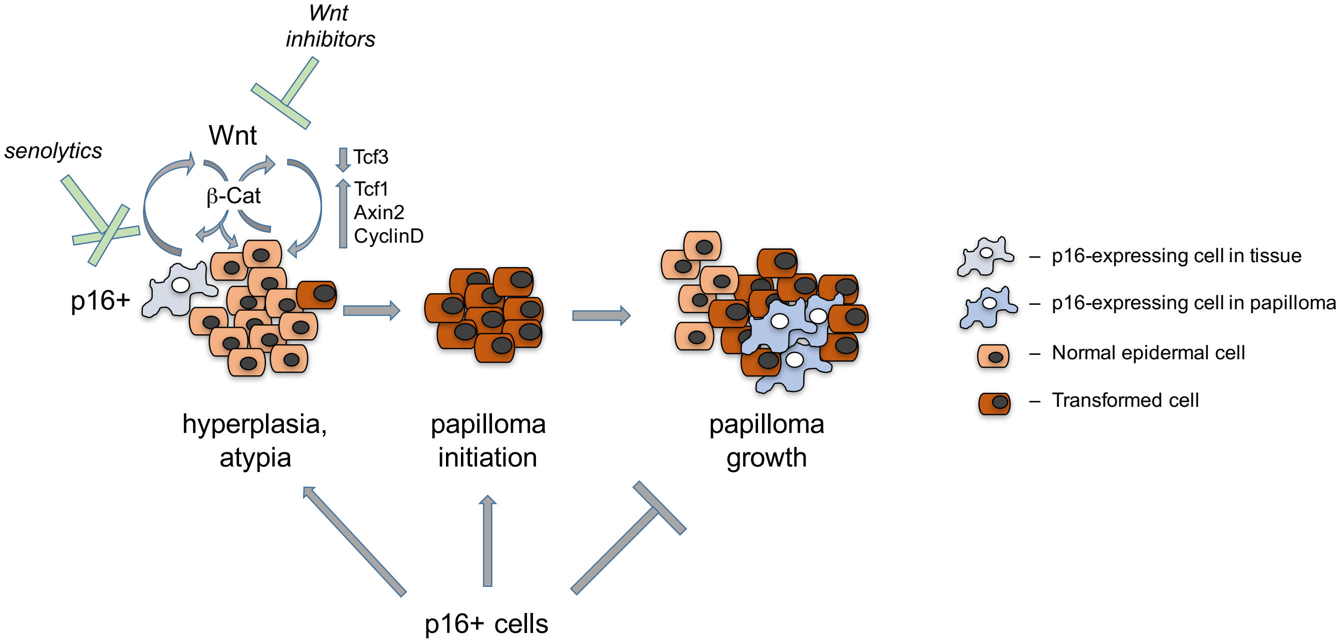

Supplementary Figure 7. Diagram depicting the consequences of p16 activity in the epidermis. p16$^+$ cells (in grey) activate Wnt ligands, stimulating Wnt-b-catenin pathway activation and further Wnt secretion in neighboring cells (in pink), leading to hyperproliferation, hyperplasia and atypia. This can stimulate a neighboring cell bearing an oncogenic driver mutation (in red) to expand and initiate a papilloma. p16$^+$ cells (in blue) within the papillomas limit lesion growth. The hyperplasia promoting activity of p16$^+$ cells can be suppressed by Wnt inhibitors or by senolytic drugs. The overall promoting and inhibiting effects of p16$^+$ cells on these processes are indicated by the arrows on bottom.

Supplementary Table 1

qRT-PCR primers used in the study:

Gene	Forward	Reverse
Axin2	AAGATCACAAAGAGCCAAAG	GAAAAAGTAGGTGACAACCAG
Bmp4	GAGGAGTTTCCATCACGAAGA	GCTCTGCCGAGGAGATCA
Ccnd1	AACACTTCCTCTCCAAAATG	GAACTTCACATCTGTGGC
Ccnd2	ATGATGAAGTGAACACACTC	CTTTGAGACAATCCACATCAG
Ccnd3	ACTTCCTGGCCTTGATTC	AAAGGTGTAATCTGTAGCAC
Cd44	AGCCCCTCCTGAAGAAGACT	ACTCGCCCTTCTTGCTGTAG
ΔNp63	TGCCCAGACTCAATTTAGTGA	GAGGAGCCGTTCTGAATCTG
FoxM1	AGGCTGGAAGAACTCTATTC	CTTGGTCCAATGTCAAGTAG
Fzd2	ACATCGCCTACAACCAGACC	CGGGTAGAACTGATGCACCT
Fzd7	ATCGCCTACAACCAGACCAT	CGGGTGCGTACATAGAGCAT
Hprt1	GTTAAGCAGTACAGCCCCAAA	AGGGCATATCCAACAACAAACTT
Lef1	AAGAAATGAGAGCGAATGTC	CTAGTTCATAGTATTTGGCCTG
Lgr6	CTGATGCACCTGAAGCTCAA	ACAGCACTGGTAGGCGTAGG
Lmnb1	GGGAAGTTTATTCGCTTGAAGA	ATCTCCCAGCCTCCCATT
p21	ACCTGATGATACCCAACTAC	CTGTGGCACCTTTTATTCTG
Ppia	CGCGTCTCCTTCGAGCTGTTTG	TGTAAAGTCACCACCCTGGCACAT
Sox9	CTCATTACCATTTTGAGGGG	AAAATACTCTGGTTGCAAGG
Sox21	CTGGGCAGCCTTACTCTGATTGT	CTTTCCACGCGTTCGTAAACTG
Tcf7	AGGCCAAGTACTATGAACTG	TCTTCTTTCCGTAGTTATCCC
Tcf7l1	GCAGCTGTCACAGACACAGTC	CAGGGCTATCACAAGGCTTC
Tcf7l2	TAAATCCCGGGAAAGTTTGG	GGGATCATGATGAAGGGGTA
Wnt3	GAGAAACGGAAGGAGAAATG	CTAGATCCTGCTTCTCATGG
Wnt3a	TTGGAACTGCACCACCGT	GAGCGTGTCACTGCGAAAG
Wnt4	AGGATGCTCGGACAACATCG	CGCATGTGTGTCAAGATGGC
Wnt5a	AATTCTTGGTGGTCTCTAGG	CAGAGTTTCTTCTGTCCTTG
Wnt6	TGTCAGTTCCAGTTCCGTTTC	AAACACGAAAGCTGTCTCTCG
Wnt9a	ACACCTGGACGACTCTCCC	CTTGTCACCACACGACTCTGT
