# Supplementary material for: Chronic expression of p16INK4a in the epidermis induces Wnt-mediated hyperplasia and promotes tumor initiation
Source: Nat Commun. 2020 Jun 1;11:2711. doi: 10.1038/s41467-020-16475-3 (PMC7264228; doi:10.1038/s41467-020-16475-3)
Supplement: Supplementary file 2 — Description of Additional Supplementary Files [file 41467_2020_16475_MOESM2_ESM.docx]

**File Name: Supplementary Data 1**

**Description: Up- and down-regulated genes in p16-expressing cells.** Genes up- and downregulated in GFP+ cells isolated from p16-expressing or from control mice after 6 months of induction. Genes were identified using DESeq2 as described in Methods section.
