# Supplementary material for: Chronic expression of p16INK4a in the epidermis induces Wnt-mediated hyperplasia and promotes tumor initiation
Source: Nat Commun. 2020 Jun 1;11:2711. doi: 10.1038/s41467-020-16475-3 (PMC7264228; doi:10.1038/s41467-020-16475-3)
Supplement: Supplementary file 4 — Reporting Summary [file 41467_2020_16475_MOESM4_ESM.pdf]

nature research

Corresponding author(s): Ittai Ben-Porath

Last updated by author(s): Feb 18, 2020

Reporting Summary

Nature Research wishes to improve the reproducibility of the work that we publish. This form provides structure for consistency and transparency in reporting. For further information on Nature Research policies, see Authors & Referees and the Editorial Policy Checklist.

Statistics

For all statistical analyses, confirm that the following items are present in the figure legend, table legend, main text, or Methods section.

n/a	Confirmed	
☐	☒	The exact sample size (n) for each experimental group/condition, given as a discrete number and unit of measurement
☐	☒	A statement on whether measurements were taken from distinct samples or whether the same sample was measured repeatedly
☐	☒	The statistical test(s) used AND whether they are one- or two-sided<br>*Only common tests should be described solely by name; describe more complex techniques in the Methods section.*
☒	☐	A description of all covariates tested
☐	☒	A description of any assumptions or corrections, such as tests of normality and adjustment for multiple comparisons
☐	☒	A full description of the statistical parameters including central tendency (e.g. means) or other basic estimates (e.g. regression coefficient) AND variation (e.g. standard deviation) or associated estimates of uncertainty (e.g. confidence intervals)
☐	☒	For null hypothesis testing, the test statistic (e.g. F, t, r) with confidence intervals, effect sizes, degrees of freedom and P value noted<br>*Give P values as exact values whenever suitable.*
☒	☐	For Bayesian analysis, information on the choice of priors and Markov chain Monte Carlo settings
☒	☐	For hierarchical and complex designs, identification of the appropriate level for tests and full reporting of outcomes
☒	☐	Estimates of effect sizes (e.g. Cohen's d, Pearson's r), indicating how they were calculated

Our web collection on statistics for biologists contains articles on many of the points above.

Software and code

Policy information about availability of computer code

Data collection	Microscopic Image collection: NIS elements BR (Nikon) V. 3.0, FV10-ASW v. 4.2. FACS: FACSDiva (BD) v. 8.0.1
Data analysis	mRNA seq analysis: R: v. 3.5.2, Cutadapt v. 1.18, Tophat2 v. 2.1.1, DESeq2 v. 1.22.1. Metascape (https://metascape.org). FACS analysis: FCS Express v. 4

For manuscripts utilizing custom algorithms or software that are central to the research but not yet described in published literature, software must be made available to editors/reviewers. We strongly encourage code deposition in a community repository (e.g. GitHub). See the Nature Research guidelines for submitting code & software for further information.

Data

Policy information about availability of data

All manuscripts must include a data availability statement. This statement should provide the following information, where applicable:

- Accession codes, unique identifiers, or web links for publicly available datasets
- A list of figures that have associated raw data
- A description of any restrictions on data availability

Gene expression data included in this study is deposited at the Gene Expression Omnibus (GEO) , GSE146979, publicly available.

Field-specific reporting

Please select the one below that is the best fit for your research. If you are not sure, read the appropriate sections before making your selection.

☒ Life sciences ☐ Behavioural & social sciences ☐ Ecological, evolutionary & environmental sciences

October 2018

For a reference copy of the document with all sections, see nature.com/documents/nr-reporting-summary-flat.pdf

Life sciences study design

All studies must disclose on these points even when the disclosure is negative.

Sample size	Mouse experiments were conducted using sibling cohorts, and sample size was therefore primarily determined by litter size and genotype distribution. Where necessary several independent mouse cohorts where combined to obtain statistically sufficient n numbers. For other experiments no statistical method was used for precalculation of sample sizes, and replicate numbers were defined based on standard practices accepted for experimentation of this type, typically using 3 to 6 replicates.
Data exclusions	One mouse papilloma showing an extreme outlier gene expression profile was excluded from the expression profile comparisons, otherwise no samples were excluded.
Replication	All experiments were performed independently at least twice, and were successfully replicated. The FACS analysis of K10+ and hair follicle stem cell percentage experiment was conducted once due to validation through complementary methods. The co-induction experiment of Tcf3+p16 was conducted once due to the complexity of obtaining triple-transgenic mouse cohorts and the length of the experiment.
Randomization	All mouse experiments were conducted on sibling mouse litters which were caged together and co-treated, with no separation by genotype or other parameter. All long-term experiments were conducted on single gender mice as described, to avoid gender influence. No other experiments involved sample group assignment.
Blinding	Experiments were largely done without blinding. The potential for blinding was limited by the need to assign genotypes to animals, and by distinct features of control and experimental groups.

Reporting for specific materials, systems and methods

We require information from authors about some types of materials, experimental systems and methods used in many studies. Here, indicate whether each material, system or method listed is relevant to your study. If you are not sure if a list item applies to your research, read the appropriate section before selecting a response.

Materials & experimental systems

n/a	Involved in the study	
☐	☒	Antibodies
☐	☒	Eukaryotic cell lines
☒	☐	Palaeontology
☐	☒	Animals and other organisms
☐	☒	Human research participants
☒	☐	Clinical data

Methods

n/a	Involved in the study	
☒	☐	ChIP-seq
☐	☒	Flow cytometry
☒	☐	MRI-based neuroimaging

Antibodies

Antibodies used	Antibodies used: human p16INK4a (Abcam, ab108349, 1:1000), human p16INK4a (BD Pharmingen, 551153, 1:200), p-Rb (Santa Cruz, sc-16669-R, 1:200), Ki67 (Abcam, ab16667, 1:200), BrdU (Bio-Rad, MCA2060,1:200), Mcm7 (Santa Cruz, sc-9966, 1;200), E-Cadherin (BD Pharmingen, 610182, 1:100), K14 (Progen, GP-CK14, 1:300), K10 (Santa Cruz, sc-23877, 1:300), K5 (Progen, GP-CK5), Rb (Santa Cruz, sc-102), CyclinD1 (Abcam, ab16663), beta-catenin (BD Pharmingen, 610153, 1:300), Tcf1 (Invitrogen, MA5-14965, 1:100), Tcf3 (Santa Cruz, sc-8635, 1:100), and CD44 (eBioscience, 14-0441-82, 1:100), Vimentin (Cell Signaling, 5741, 1:200), CD3 (Bio-Rad, MCA1477, 1:200), F4/80 (Bio-Rad, MCA497, 1:200). CD34 (BD Bioscience, 562608, 1:200), Sca-1 (Biolegend, 108113, 1:300), CD49f (eBioscience, 17-0495-80,1:200), CD45 (eBioscience, 25-0451-82, 1:1500), CD140a (eBioscience, 17-1401-81, 1:300), CD31 (eBioscience, 17-0311-80, 1:300).
Validation	All primary antibodies were verified to be validated by the manufacturer for species specificity and the application used.

Eukaryotic cell lines

Policy information about cell lines

Cell line source(s)	Primary mouse keratinocytes were generated according to described procedures by Dr. Chen Luxenburg.
Authentication	No further authentication was conducted on primary mouse cells.
Mycoplasma contamination	Cells were routinely tested for mycoplasma and found mycoplasma free in all cases.
Commonly misidentified lines (See ICLAC register)	Were not used in this study.

Animals and other organisms

Policy information about studies involving animals; ARRIVE guidelines recommended for reporting animal research

Laboratory animals	The following transgenic mouse lines were crossed as indicated: tet-p16 (mixed C57Bl6 and 129sv), K5-rtTA (FVB), tet-GFP (mixed C57Bl6 and 129sv) and tet-Tcf3 (FVB). For transgene induction mice received 2mg/mL doxycycline (Bio Basic) in their drinking water starting at the age of 3 weeks, and treatment was maintained for the indicated durations. In all experiments, the experimental and control mouse groups were siblings grown and treated together. For short-term inductions (up to 2 weeks), both males and females were used; for inductions of 6 months and wound-healing assays all presented results are from male mice, whereas for papilloma formation assays only data of females is shown, due to higher average lesion formation rates. All experiments were initiated in 3 week-old mice and continued as indicated up to 6 months. Animals were housed in specific pathogen free conditions, at 20-24°C, 30-70% humidity and a 12:12 hour light-dark cycle.
Wild animals	Were not used in this study.
Field-collected samples	Not conducted.
Ethics oversight	Animal procedures were approved by the Hebrew University Animal Care and Use Committee.

Note that full information on the approval of the study protocol must also be provided in the manuscript.

Human research participants

Policy information about studies involving human research participants

Population characteristics	A total of 72 human actinic keratosis lesions, surgically resected, were collected in three medical centers: 19 cases were obtained from the laboratory of Histology-Embryology, Athens Medical School; 42 lesions from 35 patients were obtained from the University of Arizona, USA; 11 cases were obtained from the Hadassah Medical Center, Jerusalem, Israel. All slides underwent pathologist review to verify lesion type. Patients were aged 38 to 92, with equal representation of males and females. These covariates did not influence scoring.
Recruitment	All patient material available was included in the analysis, with no selection or biases introduced that may influence scoring.
Ethics oversight	Clinical sample collection and their experimental use were approved by the Internal Review Boards of the respective institutions: the Bio-Ethics Committee of Athens Medical School, in accordance with the Declaration of Helsinki and local laws and regulations, following written consent from patients; the University of Arizona Institutional Review Board protocol, with patient informed consent, and the Hadassah Medical Center Helsinki Committee, with approved waiver of patient consent for usage of fully anonymized archival specimens.

Note that full information on the approval of the study protocol must also be provided in the manuscript.

Flow Cytometry

Plots

Confirm that:

☒ The axis labels state the marker and fluorochrome used (e.g. CD4-FITC).

☒ The axis scales are clearly visible. Include numbers along axes only for bottom left plot of group (a 'group' is an analysis of identical markers).

☒ All plots are contour plots with outliers or pseudocolor plots.

☒ A numerical value for number of cells or percentage (with statistics) is provided.

Methodology

Sample preparation	For dissociation of mouse epidermal dorsal skins, tissues were shaved and excised, and adipose and muscle tissues were removed. The skin was then incubated with 0.25% trypsin solution overnight at 4°C, and the epidermis was scraped off, minced and suspended in a trypsin/PBS with 0.5% BSA solution.<br>For dissociation of mouse epidermal ear skins, tissue were obtained by digestion with 2mg/mL Dispase II (Roche Diagnostics) in PBS solution for 40 minutes at 37°C, and treated with 2mg/mL collagenase type II (Worthington Biochemicals) and 1mg/mL DNase I (Roche) in PBS with 2% fetal calf serum (FCS) for 25 minutes at 37°C. Samples were then incubated with 0.5M EDTA for 10 minutes. Cells were treated with fixation and permeabilization solution (BD Pharmingen) for K10 staining.<br>Single cell suspensions were stained later on with primary anti-mouse antibodies.
Instrument	BD FACS Aria II was used for sorting, and a Miltenyi Biotec MACSQuant Analyzer was used for flow cytometry analysis.
Software	Data analysis was done using FCS Express software (version 4)
Cell population abundance	Purity of sorted epidermal cells for profiling was determined using RT-PCR.

Gating strategy

A figure describing gating strategy sortng cells is provided in the supplementary information. Boundaries between positive and negative staining was determined depending on single stains for each antibody.

☒ Tick this box to confirm that a figure exemplifying the gating strategy is provided in the Supplementary Information.
